# Drug repurposing candidates for amyotrophic lateral sclerosis using common and rare genetic variants

**DOI:** 10.1093/braincomms/fcaf184

**Published:** 2025-05-09

**Authors:** Zachary F Gerring, Oneil G Bhalala, Liam G Fearnley, Lotta E Oikari, Anthony R White, Eske M Derks, Rosie Watson, Nawaf Yassi, Melanie Bahlo, William R Reay

**Affiliations:** Population Health and Immunity Division, Walter and Eliza Hall Institute of Medical Research, Parkville, Victoria 3052, Australia; Brain and Mental Health Research Program, Queensland Institute of Medical Research Berghofer, Herston, Queensland 4006, Australia; Department of Medical Biology, University of Melbourne, Parkville, Victoria 3052, Australia; Population Health and Immunity Division, Walter and Eliza Hall Institute of Medical Research, Parkville, Victoria 3052, Australia; Department of Neurology, The Royal Melbourne Hospital, University of Melbourne, Parkville, Victoria 3052, Australia; Population Health and Immunity Division, Walter and Eliza Hall Institute of Medical Research, Parkville, Victoria 3052, Australia; Department of Medical Biology, University of Melbourne, Parkville, Victoria 3052, Australia; Brain and Mental Health Research Program, Queensland Institute of Medical Research Berghofer, Herston, Queensland 4006, Australia; Brain and Mental Health Research Program, Queensland Institute of Medical Research Berghofer, Herston, Queensland 4006, Australia; Brain and Mental Health Research Program, Queensland Institute of Medical Research Berghofer, Herston, Queensland 4006, Australia; Population Health and Immunity Division, Walter and Eliza Hall Institute of Medical Research, Parkville, Victoria 3052, Australia; Department of Neurology, The Royal Melbourne Hospital, University of Melbourne, Parkville, Victoria 3052, Australia; Population Health and Immunity Division, Walter and Eliza Hall Institute of Medical Research, Parkville, Victoria 3052, Australia; Department of Neurology, The Royal Melbourne Hospital, University of Melbourne, Parkville, Victoria 3052, Australia; Population Health and Immunity Division, Walter and Eliza Hall Institute of Medical Research, Parkville, Victoria 3052, Australia; Department of Medical Biology, University of Melbourne, Parkville, Victoria 3052, Australia; Menzies Institute for Medical Research, College of Health and Medicine, University of Tasmania, Hobart, Tasmania 7000, Australia

**Keywords:** drug repurposing, genome-wide association studies, exome-wide association studies, stem cells, motor neurone disease

## Abstract

Amyotrophic lateral sclerosis (ALS) is a devastating neurodegenerative condition for which novel disease modifying therapies are urgently needed. Given the increasing bottlenecks in drug discovery pipelines, repurposing existing drugs for ALS may represent a path to expedite translation and improve disease outcomes. However, ALS is a heterogeneous disease for which the aetiology remains poorly characterized, complicating efforts to effectively repurpose drugs. We propose that the polygenic architecture of ALS genetic liability, which ranges from ultra-rare, high-impact variation to common frequency loci of small-individual effect, could be leveraged to prioritize drug repurposing candidates which are more generalizable to the ALS clinical population. Here, we utilize common and rare frequency ALS genetic risk with a novel approach to uncover therapeutic classes that may be prospective repurposing opportunities in ALS. The common variant-led analyses integrated both positional-based and functional gene-based tests on SNP-genotype data from a genome-wide association study of ALS and implicated mitogen-activated protein kinase signalling related downregulation through B-Raf inhibitors as a prospective target for repurposing. The rare variant-led approaches leveraged rare variant burden testing of exonic variation on whole genome-sequencing data from a subset of the common variant genome-wide association study cohort and prioritized B-vitamin related candidates, such as cobalamin and niacin. Clinical characterization of these putative repurposing opportunities revealed genetic support to existing biology for which related compounds are actively proceeding through ALS clinical studies. Moreover, leveraging transcriptomic data from ALS derived cell lines carrying a selection of pathogenic variants in genes that cause familial forms of ALS (*C9orf72*, *SOD1*, *FUS* and *TARDBP*) suggested that the action of B-Raf inhibitors may be of particular relevance to *C9orf72* carriers, whilst the signal for B-vitamin signalling related targets was strongest in *SOD1* carriers. In summary, we demonstrate the importance of considering the therapeutic actionability of both common and rare-variant mediated risk for ALS given the immense biological heterogeneity of this disorder. Future pre-clinical and clinical studies are now warranted to further characterize the tractability of these prioritized compounds.

## Teaser

Gerring *et al.* report opportunities for drug repurposing in amyotrophic lateral sclerosis by leveraging large-scale genomic datasets. Their analysis prioritized B-vitamin signalling related targets, providing genetic support for the results of a recent phase-III clinical trial in the disorder, as well as implicating a novel repurposing candidate (B-raf inhibitors).

## Graphical Abstract

**Graphical Abstract:**
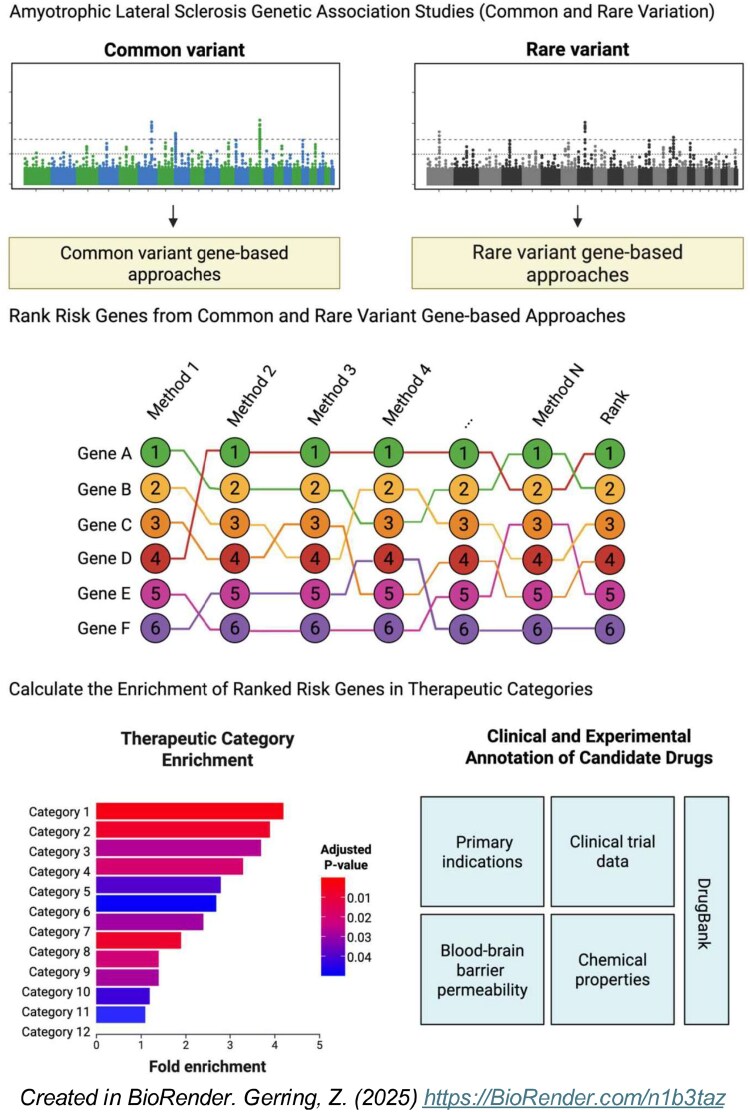

## Introduction

Amyotrophic lateral sclerosis (ALS) is a fatal neurodegenerative disease with progressive muscle weakness due to upper and lower motor neuron degeneration, as well as extra-motor presentations including autonomic impairment and cognitive dysfunction.^1^ Degeneration impacting the pyramidal tract results in eventual paralysis, with respiratory failure being the most common cause of death.^2^ A hallmark of ALS is its heterogeneity in terms of onset, presentation and clinical course—which has challenged effective therapeutic development.^3^ There is no cure for ALS, and treatments are primarily focused on managing symptoms and slowing disease progression. Two medications, riluzole and edaravone, have been approved for the treatment of ALS. Riluzole is postulated to act via glutamatergic signalling that may help to reduce motor neuron damage, whilst edaravone is an antioxidant that may protect neurons from oxidative stress. However, both medications have limited effects on disease progression and do not significantly prolong survival.

The heritability of ALS risk was established with discovery of so-called familial forms of ALS, whereby transmission of risk often follows a recognizable form of Mendelian inheritance arising from highly penetrant variation in genes such as *C9orf72*, *SOD1*, *TARDBP* and *FUS.*^2^ However, the majority of ALS cases do not exhibit this strong familial clustering and instead are often defined as sporadic. This conceptualization of ALS as sporadic is somewhat of a misnomer in the sense that ALS liability is a complex trait, which has been quantitatively demonstrated to be heritable and polygenic, with a contribution of risk along the allele frequency spectrum from ultra-rare to common.^4,5^ Genome-wide association studies (GWAS), which test for differences in allele frequencies of genetic variants between affected cases and unaffected controls, have identified common and rare variant risk alleles for ALS, as well as genes that harbour a disproportionately large burden of damaging rare variation (e.g. *NEK1*).^4^ The polygenic nature of ALS genetic risk provides an opportunity to harness this biological heterogeneity in a manner that is therapeutically relevant.

**Figure 1:**
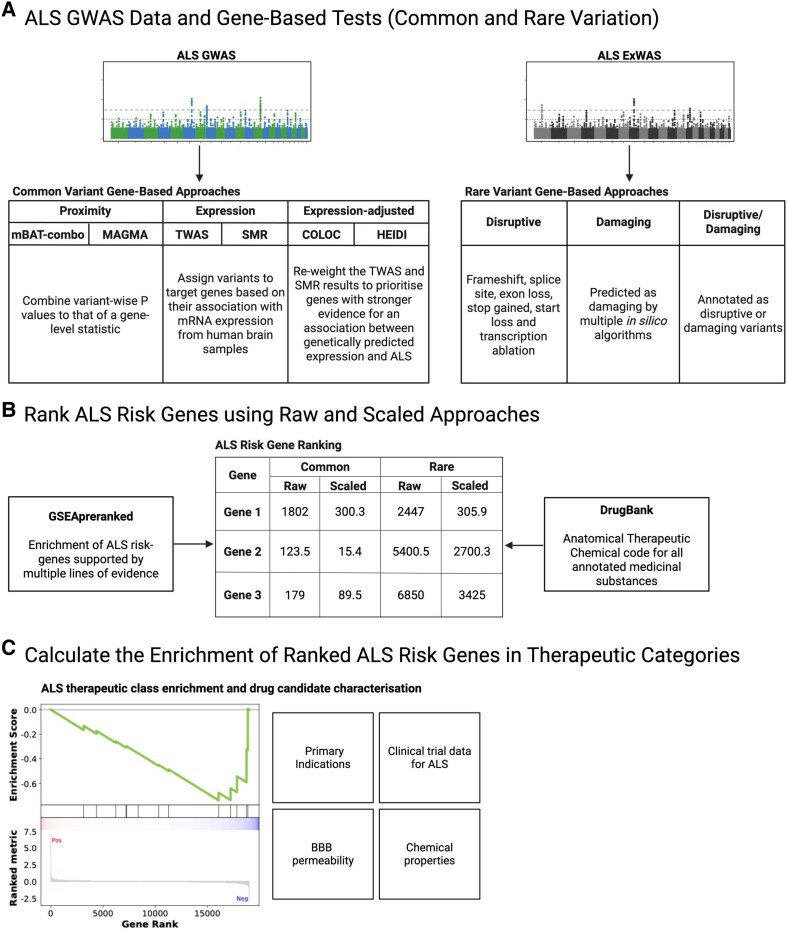
**Schematic of genetics led-drug repurposing pipeline for ALS**. (**A**) Common-frequency variants identified by GWAS of ALS were analysed using gene-based proximity (mBAT and MAGMA), expression (TWAS and SMR) and adjusted expression (COLOC and HEIDI) approaches. Low-frequency variants identified by ExWAS of ALS were classified by van Rheenen *et al.*^4^ as being either disruptive, damaging or disruptive/damaging at a gene level. (**B**) Genes identified from common- and rare-variant approaches were scaled and ranked. GSEApreranked was applied using the ranks to test enrichment amongst ATC codes for anatomic therapeutic code subclasses from DrugBank, resulting in a prioritized list of drug classes enriched for best ranked (closer to 1) genes (**C**). Created in BioRender. Bhalala, O. (2025) https://BioRender.com/x29f210.

The limited number of approved treatments for ALS and their modest effect on disease progression and outcomes underscores the need for new approaches for effectively translating drugs into clinical practice. One approach is drug repurposing, where an approved or experimental drug is tested for clinical efficacy in a different disease to its original indication.^6-9^ Computational drug repurposing provides an experimentally flexible approach by leveraging existing large-scale genomic datasets and functional annotation data to identify and characterize interactions between drugs and their target genes within a meaningful biological context. Approaches that implement computational drug repurposing have identified plausible drug candidates for a range of complex disorders and traits,^10-13^ including ALS. For example, a recent drug target enrichment analysis integrated common variant associations from GWAS with molecular data types, such as gene expression, and identified selective calcium channel blockers as repurposing candidates in ALS.^14^ Whilst this expression-based approach identifies drugs that counteract disease-associated changes in gene expression, it may discard important genetic effects that are not captured by functional gene annotation approaches and may also be confounded by the pleiotropic nature of genetic effects on mRNA expression.^15^ Furthermore, these methods can only be used currently to estimate the effect of common variants underlying disease risk. Here, we propose a powerful approach (summarized in Fig. 1) that takes into account functional and functionally agnostic gene-based approaches, allowing comparison of results across the allele frequency spectrum. Applying our pipeline to large-scale ALS genetic data supports pre-clinical and clinical evidence for therapeutics like BRAF inhibitors (BRAFi) and B-vitamin compounds.

## Materials and methods

### Study design

The principal aim of this study was to utilize large-scale genetic studies of ALS (observational studies) to inform candidates for drug repurposing. The sample size of the data leveraged here was not pre-selected, with the largest sample-size GWAS/exome-wide association study (ExWAS) at time of analysis utilized.

### Amyotrophic lateral sclerosis genetic association data

We leveraged common and rare variant association data for ALS, as extensively described by van Rheenen *et al.* in 2021.^4^ First, common variant GWAS of ALS liability was conducted via mega-analysis of >100 cohorts to assemble 27 205 ALS cases and 110 881 controls (European ancestry). There were 10 461 755 variants with ALS effect sizes available in the downloaded summary statistics. Second, rare variant burden testing of exonic variation leveraged whole genome-sequencing (WGS) from a subset of the common variant GWAS cohort (*N*_Cases_ = 6,538 and *N*_Controls_ = 2415) conducted by the Project MinE sequencing consortium,^16,17^ forthwith referred to as an ExWAS. ALS cases were included in these analyses regardless of family history, therefore, capturing both sporadic and familial forms of ALS in the GWAS/ExWAS.

### Common variant gene-based approaches

We implemented eight approaches to rank genes using common variant informed statistical methods that summarize the relationship between ALS and genetic variation and that collapse variant-level association effect sizes to individual genes (MAGMA, mBAT-combo, TWAS, colocalization weighted TWAS, SMR—brain, SMR HEIDI weighted—brain, SMR—blood and SMR HEIDI weighted—blood). A detailed description of the assumptions and statistical considerations of each approach is provided in the supplementary methods. First, we used two approaches that assign variants to genes based on physical position in the genome and then combine variant-wise *P*-values to that of a gene-level test-statistic (MAGMA v1.1.0 and mBAT-combo implemented in GCTA v1.94.1).^18,19^ Second, two methods were utilized that assign variants to target genes based on their association with mRNA expression—FUSION [transcriptome-wide association study (TWAS)] and summary-data based Mendelian randomization (SMR).^20,21^ In the FUSION TWAS approach, variants are weighted based on their association with significantly heritable mRNA expression in multivariate models trained on post-mortem cortex samples from the PsychENCODE consortium.^22^ The variant-weights from these models of genetically regulated expression (GReX) are then integrated with their association with ALS to compute signed *Z* scores that represent the association between increasing genetically predicted expression and ALS risk. In the case of SMR, individual variants strongly associated with expression are used as instrumental variables (IVs) to estimate the relationship between genetically predicted expression and ALS liability. We used both brain and blood expression-IVs for SMR.^23,24^ Expression based approaches can give rise to false positives due to the pleiotropic association of variation with the expression of multiple genes and linkage disequilibrium related confounding.^15^ As a result, we developed two novel approaches to re-weight the TWAS and SMR results to prioritize genes with stronger evidence for an association between genetically predicted expression and ALS (Supplementary Methods). These re-weighted approaches that leveraged Bayesian colocalization and the frequentist heterogeneity in dependent instruments (HEIDI) test,^21,25^ were used alongside the raw SMR/TWAS ranks to evaluate the consistency of inferred ranks.

### Rare variant gene-based approaches

Given the limited power at this sample size to estimate variant-level effects for rare loci, transcript-based burden testing of exonic variants in ALS was previously undertaken, as reported more extensively elsewhere.^4^ This involved exome-wide burden testing at two minor allele frequencies (MAF; 0.01 and 0.005), at which each of the following annotation masks was sequentially tested: disruptive, damaging, non-damaging missense, as well as the evaluation of synonymous variants. Forthwith, disruptive variation denotes those annotated as one of frameshift, splice site, exon loss, stop gained, start loss and transcription ablation; whilst damaging variants were missense loci predicted as damaging by multiple *in silico* prediction tools. Missense variants which did not meet the criteria for damaging were annotated as non-damaging. Burden tests at both MAF thresholds were also conducted using two combined annotation sets: (i) disruptive and damaging and (ii) disruptive, damaging and non-damaging missense. Burden testing was undertaken for transcripts where at least five individuals had a non-zero count of variants falling into the annotation mask considered. In our study, we considered gene-level association rather than that of transcripts for harmonization with common variant approaches, and as a result, we combined transcript-level *P*-values (derived from Firth logistic regression burden testing) into a gene-level *P*-value by leveraging the characteristics of the Cauchy distribution to guard against inflation due to covariance amongst the input *P*-values (Supplementary Text).^26^ These gene-wise *P*-values were then ranked per annotation in an ascending manner and specifically compared to that observed of synonymous variation. The rare variant ranks taken forward for further analysis via GSEApreranked were (Supplementary Table 1): disruptive (MAF <0.01), disruptive (MAF <0.005), damaging (MAF <0.01), damaging (MAF <0.005), disruptive and damaging (MAF <0.01), disruptive and damaging (MAF <0.005), disruptive, damaging, and non-damaging missense (MAF <0.01), and disruptive, damaging, and non-damaging missense (MAF <0.005).

### Combined ranks across predictors

To combine ranks across predictors (common and rare separately) we used two approaches. First, we calculated the common and rare variant median rank based on all available ranks (with a rank of ‘1’ indicating the best rank), ignoring ranks missing for a particular gene. In other words, for genes with only one rank available, this rank was reported, whilst the median calculated of *n* (range of 2 to 8) available ranks was reported for all other genes—we term this the ‘raw median’. The coefficient of variation was calculated between ranks for genes with at least five input ranks. The coefficient of variation captures variability between ranks as it is the standard deviation across ranks in a gene divided by the per-gene mean rank. A limitation of our approach is that it can highly weight genes with only one or two input ranks available if those lines of evidence are top ranking. Whilst this does not preclude the ALS risk relevance of a gene, it would be considered less confidently ranked relative to genes ranked closer to one across most of the input methods. As a result, we also implemented a ‘scaled’ median approach alongside the raw median. The scaled median is calculated by dividing the raw median by the number of non-missing ranks for that gene, such that genes with more non-missing input ranks are more highly weighted. We considered both the raw and scaled medians (for both common and rare variants) to balance the utility of these different approaches of combining evidence across inputs; however, the scaled approach is less sensitive to the influence of genes for which only a few or even one line of evidence contributes to its ranking, and therefore, is the primary method of interest.

### Enrichment of ranks amongst pharmacological therapeutic classes and biological annotations

Enrichment analyses against each ranked list of genes was performed using the GSEApreranked method [Gene Set Enrichment Analysis (GSEA)].^27^ The rank (.rnk) formatted files were generated for both raw and scaled median rank approaches for common and rare gene-based results. Gene matrix files (.gmt) were generated for Anatomical therapeutic chemical (ATC) codes level 2, level 3 and level 4 for all annotated medicinal substances in DrugBank.^28,29^ We also generated.gmt files for mechanism of action categories from the connectivity map (CMAP) and gene ontology (GO) molecular function, biological processing and cellular component annotations from the molecular signatures database (MSigDB).^30,31^ GSEApreranked was performed using the python implementation *GSEApy* v1.1.3.^32^ We used the default weighted scoring statistic with 10 000 permutations, and restricted analysis to gene sets with between 5 and 500 genes. The enrichment score (ES) indicates the degree to which the gene-set (for example, an ATC code) is represented at the higher or lower end of the ranked gene-list. We are interested in negative ES values as these indicate sets enriched for best ranked genes (i.e. closer to 1), and thus, are our ‘top’ ranked genes. In order to assess enrichment in a more meaningful way, we focus on the normalized enrichment score (NES) that accounts for factors such as gene-set size by dividing the ES by the mean ES across all permutations. False discovery rate (FDR) and Bonferroni were used for multiple-testing correction at each tail of the distribution in line with usual GSEA practice. We note that due to the irregular shape at the tails of distribution curves in GSEA, the Bonferroni corrected family wise error rate (FWER) values can sometimes be smaller than that of the corresponding FDR. Here, we considered both FDR and FWER <0.05 as strong evidence, whilst FDR or FWER <0.05 as moderate evidence.

### Integration of publicly available iPSMN RNA-seq data

We obtained publicly available data from pluripotent stem cell-derived motor neurons (iPSMN) lines across different genetic backgrounds.^33^ This resource contains 429 iPSMNs, of which 383 were derived from ALS patients and 106 from non-ALS controls. Uniformly processed and quality controlled differential gene expression data were available for four different ALS mutations (*C9orf72*, *SOD1*, *TARDBP* and *FUS*) as well as sporadic ALS. Differential expression statistics for weach gene were also available for a consolidated ‘pan-ALS’ analysis that encompassed sporadic and the four specific variant carrier lines. Differentially expressed genes were ranked by statistical significance (*P*-value) within each of the four different ALS iPSMN mutation subgroups, in addition to the sporadic ALS iPSMN subgroup and the consolidated ‘pan-ALS’ results. We generated.*rnk* files for each ranked list and ran GSEApreranked against ATC level 4 and mechanism of action.*gmt* files using the same algorithmic parameters described above.

### Clinical tractability of prospective amyotrophic lateral sclerosis repurposing candidates

We evaluated the clinical tractability of drugs within enriched ATC codes and mechanisms of action groups across several domains. Safety profiles and adverse reactions were extracted from clinical trial data and post-marketing surveillance reports (sourced from DrugBank).^29^ Primary indication, contraindication and chemical property information was extracted from DrugBank and Food and Drug Administration (FDA) labels for approved drug compounds. We calculated the properties related to Lipinski's ‘rule of five’, which incorporates information across four drug properties, including its hydrophilicity/lipophilicity (its ‘partition coefficient’ [log*P*]), molecular weight, and the number of hydrogen bond donors and acceptors, to assess the likelihood of absorption (i.e. whether a drug is orally active in humans) for each drug repurposing candidate. We also assessed the predicted probability of blood–brain–barrier permeability using the prediction of absorption, distribution, metabolism, excretion and toxicity (ADMET) features generated by the admetSAR tool and reported in DrugBank.^34^

### Statistical analyses

We provide a brief summary forthwith of the statistical methodology deployed here, with full details provided in each respective section of the methods. Sample size was not pre-specified *a priori* as this was a discovery driven study that sought to use the largest sample-size ALS genetic datasets available. The central genetic datasets utilized in this study were a GWAS and a burden-based ExWAS, both of which were performed using logistic regression, as described in the source publication. We deployed Cauchy aggregation to combine the ExWAS transcript-level *P*-values (each variant mask individually) into a combined gene-based *P*-value for subsequent ranking. Common-variant gene-based rankings deployed four separate statistical methods (MAGMA, mBAT-combo, TWAS and SMR—Supplementary Methods). Correlations between the gene-based ranks were estimated using Spearman's rho. The gene-wise coefficient of variation between input ranks was calculated via dividing the mean rank by the standard deviation of ranks. We implemented the GSEApreranked method with default weightings to identify gene-sets enriched at the lower or higher end of ranks, with FDR and FWER used for multiple-testing correction. Given the small-sample size and extensive ties between ranks, we used Kendall's tau to estimate correlations between the ATC code ranks.

## Results

### Ranking of amyotrophic lateral sclerosis risk genes using common and rare variant led approaches

**Figure 2:**
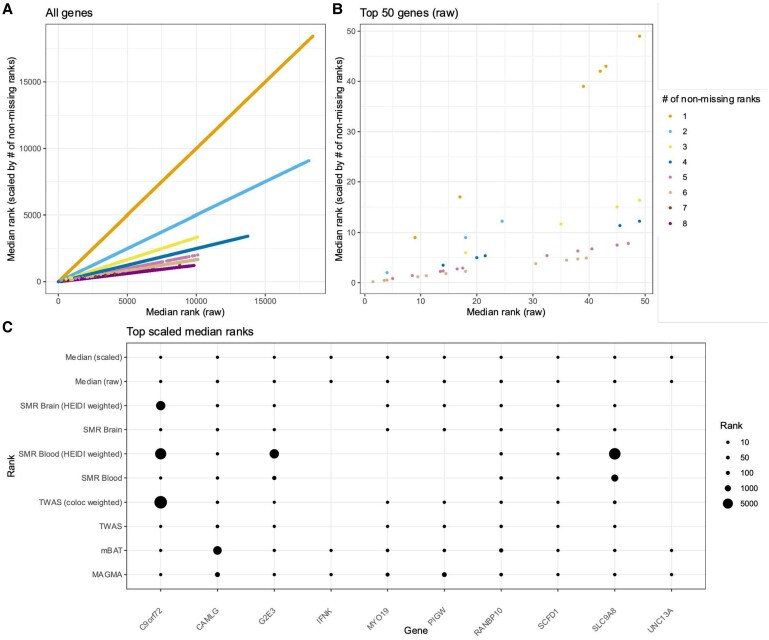
**Common variant led ranking of genes using ALS GWAS data**. In this approach, eight different gene-based methods are leveraged to rank genes (each point a rank) in an ascending fashion with the common variant GWAS as input (27 205 ALS cases and 110 881 controls), such that the top gene is ranked ‘1’. In panel (**A**), the *x*-axis denotes the median rank across all gene-wise input ranks, whilst the *y*-axis denotes the scaled median rank that upweights genes with a greater number of non-missing input ranks. This scatter plot visualizes the relationship between the raw and scaled median rank, with points coloured by the number of non-missing ranks per-gene (no statistical comparison performed between raw and scaled metrics). The same relationship is plotted in panel (**B**) for just the genes ranked in the top 50 using the raw median—further demonstrating that genes with only a small number of input ranks are penalized compared to those with less missingness, and therefore, a more representative median estimate. (**C**) Genes with the top 10 scaled median ranks are visualized in terms of their input rank for each method, with whitespace denoting a missing rank for that method.

We first implemented a novel pipeline that uses different approaches to estimate gene-level associations with ALS for common and rare variation, separately. The summary characteristics of the common variant ranks (*N*_Genes_ = 19,888; Supplementary Table 2 and Supplementary Figs 2 and 3) are described in the supplementary text. In Fig. 2, we visualize the raw median common variant ranks, as well as how this relates to the scaled median ranks, which upweight genes with more non-missing input ranks. This reveals that the key ALS risk gene *C9orf72,*^35^ was a top ranked gene across positional-based (MAGMA and mBAT) and expression-based (TWAS and SMR) approaches; however, is heavily penalized by the adjusted expression approaches like colocalization adjusted TWAS—which is likely due to the lead variant tagging a repeat expansion, which is likely to have multiple founder events, as characterized previously.^4^ In contrast, the myosin gene *MYO19*, and sec1 family domain gene (*SCFD1*), involved in autophagy, which are both biologically plausible ALS risk genes,^36,37^ are highly ranked by all approaches, including the colocalization adjusted TWAS, suggesting a potential shared causal variant between ALS and the expression of these two risk genes. Overall, this highlights the value of including different approaches for gene-based association testing to capture the advantages and disadvantages of each of their underlying assumptions, especially in the context of atypical (non-SNV) genetic contributors.

Summary characteristics of the rare variant ranks are also outlined in the supplementary text (*N*_Genes_ = 17,489; Supplementary Figs 4–6 and Supplementary Table 3). We emphasize that the interpretation of individual genes is much more difficult for rare variants given the comparative lack of statistical power in the ExWAS compared to that of common variants. Instead, we focused on these results in terms of the relative enrichment of highly ranked genes in therapeutic classes, as discussed in a proceeding section of the study.

The correlation between common and rare variant median ranks was small in magnitude but statistically significant—median common versus rare (raw): *ρ* = 0.02, *P* = 5 × 10^−3^, median common versus rare (scaled): *ρ* = 0.05, *P* = 1 × 10^−11^. Genes in the 10% of ranks closest to one also exhibited a lower median shift if that gene had a higher probability of haploinsufficiency [probability of loss of function intolerance (pLI) > 90%, Supplementary Fig. 7]. The gene encoding NIMA-related kinase 1 (*NEK1*) appeared as a gene of interest highlighted using both common variant and rare variant led approaches (Supplementary Fig. 8), which previous experimental work suggests may be therapeutically actionable.^38^ As outlined in the supplementary text, we found that *NEK1* exists within gene-sets relevant to the polygenic signal for ALS and is linked to ALS relevant clinical endpoints upon scanning an association between genetically predicted *NEK1* expression and the human clinical phenome (Supplementary Fig. 7 and Supplementary Table 4). Finally, we also found that a subset of genes suggested to cause ‘familial’ forms of ALS were prioritized using either the common or rare variant ranks (Supplementary Fig. 9).

### Leveraging common and rare variant data reveals evidence for drug repurposing candidates in amyotrophic lateral sclerosis

Gene based ranks were utilized to prioritize repurposing candidates for ALS. The focus of this study was on overall enrichment within drug classes and mechanism of action groupings, which is likely more informative for a complex, heterogeneous phenotype like ALS. However, we do report five potential targets in the 5% of ranks closest to one (scaled median) for both common and rare-variant led approaches that are annotated using the target central resource database as either targeted by an approved drug or displaying strong small molecule binding affinity (Supplementary Fig. 10).^39^ These genes were as follows: *NEK1*, *MAPT*, *CTRL*, *DEGS1* and *TSHR*. Notably, both the *NEK1* and *MAPT* findings support previous pre-clinical data exploring microtubule stabilising compounds in ALS.^38^

**Figure 3:**
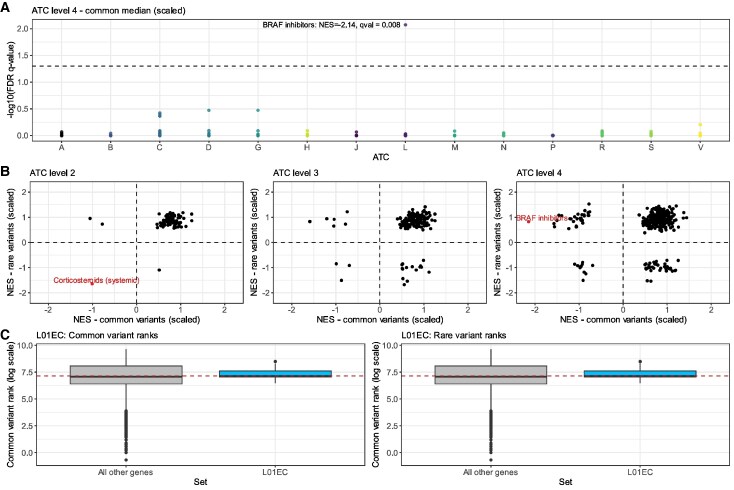
**Enrichment of ALS gene-ranks amongst ATC code drug classes**. (**A**) Manhattan-like plot that visualizes the strongest enrichment found in therapeutic category related analyses—an enrichment of best ranked (closer to one) common variant led genes in the level 4 ATC (each point an ATC code) B-raf (BRAF) inhibitors (ATC code = L01EC). Each point represents the −log10 transformed FDR *q*-value of enrichment is plotted, grouped by overall ATC code level 1 category on the *x*-axis. The ATC level 1 codes are A = Alimentary tract and metabolism, B = blood and blood forming organs, C = cardiovascular system, D = dermatologicals, G = genito-urinary system and sex hormones, H = systemic-hormonal preparations (excl. sex hormones and insulin), J = anti-infective (systemic), L = antineoplastic and immunomodulating agents, M = musculoskeletal system, N = nervous system, P = antiparasitic, insecticides and repellants, R = respiratory system, S = sensory organs and V = various. The dashed horizontal line represents FDR *q* < 0.05. (**B**) Scatterplot with each point denoting the rare and common variant NES from gene-set enrichment analysis (GSEApreranked) for each ATC category level (left to right, level 2, level 3 and level 4). Our categories of interest are those with NES <0 as this represents an enrichment of genes ranked closer to 1. Common variant led GSEApreranked results (NES) are denoted on the *x*-axis, and rare-variant led NES on the *y*-axis. Rare variant ranks derived from ExWAS with sample size of 6538 ALS cases and 2415 controls, whilst common-variant ranks derived from genome-wide association study (27 205 ALS cases and 110 881 controls). (**C**) Distribution of common variant (left) and rare variant (right) ranks, respectively, within the BRAFi ATC code (natural log scale) relative to that of all remaining genes that were ranked. Each point denotes the rank of a gene in that ATC code set. The GSEApreranked derived enrichment of common variant ranks was *P* < 0.0001 (lowest *P*-value that can be achieved with 10 000 permutations) with a *q*-value of 0.0085. Conversely, there was no significant enrichment of rare variant ranks closer in the BRAFi ATC code (*P* = 0.794).

We then used common and rare median gene ranks (scaled) to prioritize ATC groups (level 2, level 3 and level 4, respectively) in which there was a significant enrichment of best ranked genes, that is, genes ranked closer to ‘1’ (Fig. 3; Supplementary Tables 5–10). One code each was uniquely enriched with the best ranked genes after application of multiple-testing correction for common and rare median (scaled) ranks, respectively (Bonferroni corrected *P*-value <0.05 and FDR *q*-value <0.05). The most significant enrichment after multiple-testing correction was observed in the ATC level 4 code L01EC [B-Raf serine–threonine kinase (BRAF) inhibitors] for the common median ranks (scaled): NES = −2.14, *q* = 8.5 × 10^−3^ (Fig. 3). Interestingly, this enrichment was not seen for the two parent ATC codes of L01EC protein kinase inhibitors (L01E, level 3) and antineoplastic agents (L01, level 2), suggesting some specificity for this therapeutic class relative to other kinase inhibitors and antineoplastic agents in general. The other significant enrichment after multiple-testing correction was found for the rare median ranks (scaled) amongst targets in the level 2 ATC code H02 (corticosteroids): NES = −1.64, *q* = 0.034. In this instance, three child terms of this H02 code were also nominally enriched with top ranked rare variant genes but did not survive multiple-testing correction: corticosteroids for systemic use (H02A, level 3), antiadrenal preparations (H02C, level 3) and anticorticosteroids (H02CA, level 4). These corticosteroid findings will be discussed further later in the manuscript as an enrichment for both corticosteroid and anticorticosteroid mechanism of action is complex to interpret. At ATC level 3, we also found suggestive evidence for antihypertensive compounds using the common variant led approach, in support of previous work that prioritized selective calcium channel blockers as ALS repurposing candidates.^14^ Specifically, the strongest level 3 signals were found for two diuretic related codes (C03E [diuretics and potassium-sparing agents in combination]: NES = −1.59, *q* = 0.11 and C03C [high-ceiling diuretics]: NES = −1.59, *q* = 0.22).

We also explored the consistency of the ATC enrichments observed for common and rare variant rank led results (Fig. 3B). There was a significant positive correlation between the estimated NES for level 3 (Kendall's τ = 0.17, *P* = 7.2 × 10^−4^) and level 4 (τ = 0.1, *P* = 6.1 × 10^−3^) codes, whilst no significant correlation was observed for the least specific code grouping (level 2: τ = 0.115, *P* = 0.12, Fig. 3B). We note that these correlations are largely driven by the ATC codes with enrichment for ranks with larger values away from one, and thus, with no strong evidence for an association with ALS (NES >0). Using the raw median weights that were not scaled for the number of inputs also supported BRAFi (NES = −1.86, *q* = 0.16) and corticosteroids (NES = −1.67, *q* = 0.04) with similar NES estimates, although statistical significance was marginally attenuated for BRAFi. We note that the most significant therapeutic enrichment seen for BRAFi using common-variant led ranks was not revealed using a rare-variant led approach (Fig. 3C), whilst there was some nominal consistency in the common variant signal relative to that found for rare-variants in the corticosteroids level 2 code (Fig. 3B).

**Figure 4:**
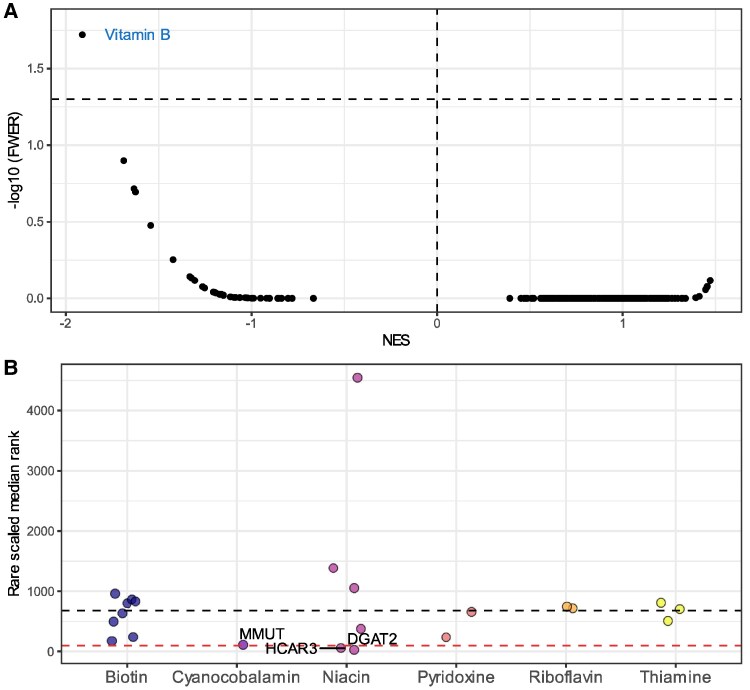
**Rare-variant led enrichment within drug mechanism of action groups**. (**A**) Volcano plot that visualizes the GSEApreranked derived enrichments within mechanism of action groups using rare-variant scaled median ranks as input (GSEApreranked algorithm with default rankings). Each point denotes the GSEApreanked results for a mechanism of action group tested. Rare variant ranks derived from ExWAS with sample size of 6538 ALS cases and 2415 controls. A NES < 0 denotes an enrichment of best ranked (closer to one) genes. The −log_10_ transformed Bonferroni correction derived FWER is plotted on the *x*-axis, with the dotted horizontal line denoting a Bonferroni corrected *P*-value <0.05. (**B**) Each point represents the scaled median rare-variant rank of specific B-vitamin targets that compromise the vitamin B mechanism of action group. The black horizontal line denotes the median rank amongst the entire mechanism of action group, whilst the red-dotted horizontal line indicates genes ranked ≤100.

As an orthogonal therapeutic enrichment analysis, we applied this same GSEApreranked approach to the mechanism of action groups sourced from CMAP (Supplementary Tables 11 and 12). We note here that BRAFi were not available in these analyses as a distinct CMAP mechanism of action grouping. We did not find any common variant enrichment with any single mechanism of action group that surpassed multiple-testing correction. Using rare variant ranks, we found evidence of enrichment amongst the ‘vitamin B’ mechanism of action gene-set—NES = −1.91, *q* = 0.065, FWER *P* = 0.02 (Fig. 4A). We note here that this is an example of FDR correction being more conservative than that of Bonferroni (FWER), which arises due to the irregular tails of the NES distribution, and that the enrichment is only suggestive considering an FDR threshold of 5%. However, this finding broadly supports previous phase III, placebo-controlled RCT data for high-dose methylcobalamin (‘active’ form of vitamin B_12_), as outlined further in the discussion.^40^ This enrichment was not seen using common variant approaches. Dissection of the individual B-vitamin targets within the set revealed this enrichment signal has an important contribution from the niacin receptor *HCAR3* and the lipid synthesis related gene *DGAT2* that niacin is known to target (Fig. 4B). The Vitamin B_12_-dependent enzyme *MMUT* was also relatively lowly ranked; with directional consistency amongst genes annotated to the level 3 ATC code B03B (vitamin B_12_ and folic acid, NES = −1.18, *q* = 0.6), although this was not significant. Moreover, these data may suggest generalized relationship to physiology linked to B-vitamins relevant to ALS pathogenesis, such as redox homeostasis.^41^ Overall, we found that the therapeutic significance of common and rare variant led approaches revealed quite distinct druggable biology in terms of specific compound groupings and mechanisms of action. We also considered broad common and rare variant rank enrichment amongst gene-ontology related gene-sets (Supplementary Tables 13–18). Whilst no gene-sets survived multiple-testing correction, which is highly penalized by the large number of gene-sets tested, the lowest common-variant NES value (greatest enrichment of ranks towards one) found for GO biological processes was *‘negative regulation of protein kinase activity by regulation of protein phosphorylation*.’

### Exploring the therapeutic tractability genetically informed amyotrophic lateral sclerosis repurposing candidates

In the proceeding section, we further explore the therapeutic tractability of our strongest prioritized compounds from either the common or rare-variant led analyses (Table 1). Orthogonal support for the potential utility of BRAFi was investigated by leveraging previous work that characterized transcriptomic signatures of ALS versus control induced pluripotent stem cell-derived motor neurons (iPSMN) lines across different genomic backgrounds (‘familial’ ALS variant carriers and ‘sporadic’), as well as a ‘pan-ALS’ combined analyses (Supplementary Figs 11 and 12).^33^ We found that top ranked genes (ranked in descending order by differential expression statistical significance) were enriched amongst the genes within the L01EC (BRAFi) level 4 code in the ‘pan-ALS’ pooled iPSMN analyses (*q* = 0.042, Supplementary Fig. 10). Considering the individual iPSMN genetic backgrounds, only the *C9orf72* carrier iPSMN data showed suggestive enrichment after multiple-testing correction (*q* = 0.055), which is notable given the relationship between *C9orf72* and BRAF signalling related biology like proteostasis.^42^ This connection between BRAFi and *C9orf72* is also supported by previous analyses on these same datasets which predicted that Mitogen-Activated Protein Kinase (MAPK) signalling was upregulated with strongest magnitude in the *C9orf72*-carrier iPSMN lines.^33^

**Table 1:** Description and clinical tractability related information for key genetically prioritized ALS repurposing candidates from this study

	Vemurafenib	Dabrafenib	Encorafenib	Vitamin B3 (Niacin)	Vitamin B12 (Methylcobalamin)
Selected primary indications	Unresectable or metastatic melanoma with BRAF V600E mutation, Erdheim–Chester disease	Unresectable or metastatic melanoma with BRAF V600E mutation non-small cell lung cancer, and anaplastic thyroid cancer	Unresectable or metastatic melanoma with BRAF V600E or V600 K mutations, metastatic colorectal cancer with BRAF V600E mutation	Severe triglyceridemia, vitamin deficiency	Pernicious anaemia, atrophic gastritis, drug toxicity reduction and others^a^
Completed or ongoing clinical trial for ALS	No	No	No	NCT04562831^b,c^ NCT05095571^b,c^ NCT03489200^d^ NCT04244630^c,d^	NCT03548311^d^ NCT00444613^d^
Chemical properties					
log*P*^e^ ≤5	Yes (4.95)	No (5.44)	Yes (4.16)	Yes (0.147)	Yes (1.897)
Molecular weight (Da) < 500	Yes (489.92)	No (519.56)	No (540.01)	Yes (123.11)	No (1344.38)
H-bond acceptors ≤10	Yes (4)	Yes (6)	Yes (7)	Yes (3)	No (13)
H-bond donors ≤5	Yes (2)	Yes (2)	Yes (3)	Yes (1)	No (9)
ADMET calculated BBB permeability [0–1]^f^	0.745	0.7232	−	0.9648	0.8526

^a^See FDA-approved indications for full list; ^b^Trial ongoing at time of writing; ^c^Combination of nicotinamide with other antioxidants; ^d^Trial completed; ^e^Partition coefficient; log*P* <0 compound is more hydrophilic, log*P* >0 compound is more lipophilic; Da, Daltons (1 Dalton = 1 g/mol); ^f^Blood–brain–barrier (BBB) permeability probability value (between 0 and 1) from ADMET. A molecule is more likely to pass the BBB if the probability value is closer to 1.^34^

Using rare variant ranks and a different therapeutic category based annotation system (CMAP mechanism of action), allowed us to capture a rare-variant signal enrichment within targets of B-vitamins, which are not encompassed by a single consolidated ATC code. Leveraging the transcriptomic signatures of the different iPSMN lines utilized above (Supplementary Fig. 1^1^), we found here that the strongest enrichment of targets with the B-vitamin mechanism of action set was observed for *SOD1* (superoxide dismutase 1) pathogenic variant carriers (NES = −2.25, *q* = 3.3 × 10^−3^). Some evidence was also seen for an enrichment of lowly ranked transcripts (more significantly differentially expressed genes) in this mechanism of action gene-set for *C9orf72* carriers (*q* = 0.052) and the ‘pan ALS’ combined analysis (*q* = 0.10), although neither survived multiple-testing correction.

We assessed the clinical tractability in relation to ALS of the three currently available BRAFi (vemurafenib, dabrafenib and encorafenib), as well as vitamin B_3_ (niacin) and vitamin B_12_ (specifically methylcobalamin to align with the previous phase-III trial) related compounds, using data from DrugBank and other publicly available resources, such as the FDA. We note for clarity that cyanocobalamin is the more widely available form of vitamin B_12_ supplementation, with methylcobalamin differing from cyanocobalamin as there is a methyl rather than cyano group bound to the central cobalt atom. The three BRAFi have overlapping adverse reactions, including the possibility of new malignancy formation, arrhythmias, cutaneous reactions, uveitis and haemorrhage. Whilst there are no absolute contraindications for these therapies, appropriate use and dosing in the presence of adverse reactions is relative to individual patients and their co-morbidities, as well as their indications for treatment (Supplementary Table 19). Moreover, it is likely that the doses used outside of oncology will be significantly lower than those indicated for aggressive malignancies. There are no known completed or ongoing trials for the use of BRAFi in ALS, although a clinical trial for trametinib, a MEK1/MEK2 inhibitor which is downstream of BRAF, was recently completed (NCT04326283), although outcomes are yet to be reported. Vitamins B_3_ and B_12_ related compounds have relatively few side effects at recommended doses but can be associated with adverse effects, such as gastrointestinal symptoms and fatigue at higher concentrations (Supplementary Table 19). Vitamins B_3_ and B_12_ also have a small number of contraindications (e.g. hepatic dysfunction for vitamin B_3_, Supplementary Table 19). A number of clinical trials are ongoing or have recently completed at the time of writing for these vitamins, or their derivatives, including in combination with other compounds (e.g. antioxidants, Table 1). The chemical properties of vemurafenib and vitamin B_3_ (i.e. molecular-weight, aqueous solubility, and number of donor/acceptor hydrogen bonds) suggest they are more favourable for oral activity using Lipinski's rule of five criteria,^43^ although dabrafenib, encorafenib and methylcobalamin are available as oral formulations despite this, with the previous trial data in ALS using a intramuscular methylcobalamin formulation. Furthermore, vemurafenib, dabrafenib, methylcobalamin and vitamin B_3_ are all likely to pass the BBB based on the ADMET score (with values ranging from 0 to 1, with 1 indicating a high probability of BBB passage, Table 1).

## Discussion

In this study, we leveraged the genetic architecture of ALS as derived from hypothesis-free genome and exome-wide association studies to prioritize repurposing candidates for which further pre-clinical and clinical consideration is warranted. Specifically, we found that a common variant approach supported BRAFi as putative ALS repurposing candidates. Interestingly, applying our repurposing pipeline using rare-variants revealed different biology—for instance, an enrichment amongst targets of B-vitamins that supports previous RCT data. In both instances, BRAFi and B-vitamin related targets were further supported using data from ALS iPSMNs profiled with RNAseq.

**Figure 5:**
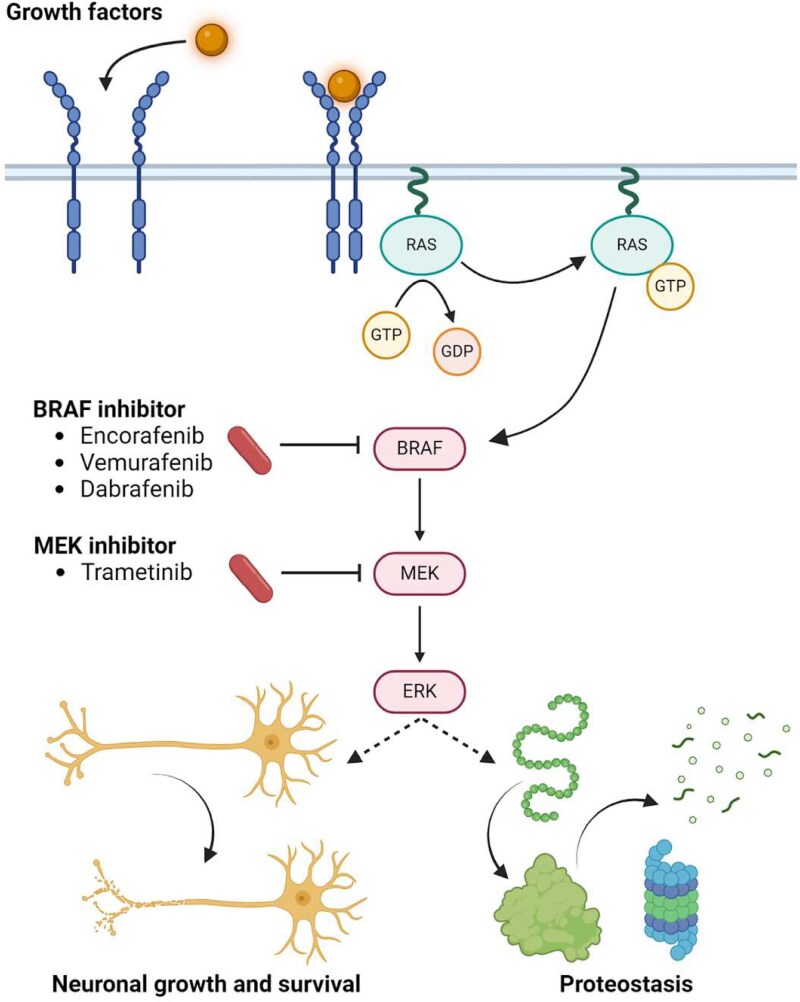
**Schematic of the biological context of BRAFi as a repurposing candidate in ALS**. The RAS/RAF/MAPK pathway mediates the transduction of extracellular signals to the cell nucleus via a phosphorylation signalling cascade, where target genes are activated for several processes in all cell types, including cell proliferation, differentiation and growth and apoptosis and autophagy. Somatic mutations in the RAF/MAPK pathway are present in many human cancers, such as the BRAF V600E mutation in malignant melanoma. Several antineoplastic drugs have been developed to target signalling molecules in this pathway, and include the BRAFi encorafenib, vemurafenib and dabrafenib, and the MEK inhibitor trametinib. Broadly, these drugs decrease ERK activation and thereby inhibit excessive cell proliferation and other dysregulated cellular processes. In particular, the effect of these compounds on proteostasis may be of particular relevance to the pathophysiology of ALS. Generated with BioRender.com. BRAF, B-raf; ALS, amyotrophic lateral sclerosis; RAS/RAF/MAPK, rat sarcoma/rapidly accelerated fibrosarcoma/mitogen-activated protein kinase; MEK, mitogen-activated protein kinase kinase. Created in BioRender. Gerring, Z. (2025) https://BioRender.com/s95p112.

BRAFi have emerged as important antineoplastic compounds informed by precision medicine—that is, in advanced cancers which somatically carry *BRAF* variants that constitutively activate signalling, most commonly a point-missense valine to glutamic acid substitution at the 600th residue (often referred to as V600E).^44^ BRAFi can be indicated as a monotherapy or in combination with MEK1/MEK2 inhibitors (MEKi) like trametinib given the ability of tumours to become resistant to inhibition of MEK or BRAF signalling alone, as well as the properties of BRAF-V600E that allow stimulation of Ras-Raf-MEK-ERK signalling (RAS/RAF/MAPK) as an active monomer which is more efficiently targeted by available BRAFi.^44,45^ However, it has been shown that current generation BRAFi do have an impact on BRAF signalling without activating variants like V600E, albeit to a much lesser extent.^46,47^ Mechanistically, the primary potential utility of BRAFi in ALS may arise through the influences of its downstream signalling consequences on the cellular proteostasis network, as well as overall neuronal survival (Fig. 5).

Proteostasis broadly refers to the maintenance of the production and degradation of intracellular protein. A significant body of evidence suggests the dysregulation of this process contributes to motor neuron degeneration in ALS and beyond, as reviewed previously.^48^ For example, the key ALS risk gene *C9orf72* is implicated to regulate autophagy-lysosome related biology that if disrupted contributes to toxic protein aggregation.^49,50^ This is notable given that the signal for BRAFi targets was strongest in our GSEApreranked analyses of differential expression RNAseq-based data from *C9orf72* carrier iPSMNs. Whilst we believe that BRAFi should be subjected to further pre-clinical investigation as a generalized therapy for ALS, these data suggest that further specific investigation of the interplay between *C9orf72* and BRAF signalling is warranted. Oncogenic aberrant BRAF signalling induced by the V600E somatic variant has been shown to impair autophagy.^51-53^ The MEKi trametinib, which targets MEK1/MEK2 that sit downstream from BRAF within the RAS/RAF/MAPK pathway (Fig. 5), has received some attention as a repurposing candidate for ALS. Murine data has suggested that trametinib can have neuroprotective properties through autophagic lysosomal activation,^54^ which has proceeded to an early stage clinical trial in ALS (NCT04326283). In our study, we could not test the MEKi ATC code specifically in the GSEApreranked analysis because there were an insufficient number of target genes in the gene set. Inspecting the ALS common and rare variant led-ranks of the genes encoding MEK1 and MEK2 did not show strong genetic support for those particular targets (Supplementary Tables 2 and 3). However, recent data leveraging transcriptomics and proteomics from post-mortem brain samples and rodent models carrying known key ALS genes has supported that trametinib, and RAS/RAF/MAPK inhibition more broadly, as an ALS therapeutic candidate.^55^ In other words, we only found genetic support for BRAFi as opposed to downstream MEK inhibition but existing literature supports that also targeting MEK may be therapeutically useful.^55^

Additional support for the role of BRAF signalling arises from studies that investigated the neuronal consequences of somatically acquired BRAF V600E variants, including evidence that links such activating variants to the ALS-relevant pathologies of hyperexcitability and neurodegeneration.^56,57^ Interestingly, pre-clinical data also suggests that somatic mosaicism of V600E in erythro-myeloid progenitor lineages give rise to microglia-driven RAS/RAF/MAPK activation that can result in neurodegeneration relevant phenotypes.^58^ We assert that the dual-inhibition (MEKi + BRAFi) should be also considered given the synergistic impacts on RAS/RAF/MAPK signalling. However, we caution that there are some important potential drawbacks to the use of these antineoplastic agents in ALS, including toxicity related concerns and the feasibility of longer-term treatment. Although, much lower dosages than utilized in oncology are more likely to be better tolerated, which is reflective of previous on-target and off-target repurposing of antineoplastic compounds for indications outside of oncology. Future work should in parallel investigate the prospect of more selective central nervous system delivery through approaches, including liposomal encapsulation. Another important consideration is the potential paradoxical feedback-driven upregulation of RAS/RAF/MAPK signalling that has been shown experimentally of some BRAFi in certain contexts,^59^ although this requires additional interrogation outside of oncology and in ALS relevant cell-types like motor neurons. This phenomenon can also be rescued by co-treatment with MEKi. Moreover, it is possible that the influence of BRAFi on ALS pathogenesis may extend to their off-target promiscuity to other kinases, which is reflected by the >10 targets recorded in DrugBank besides BRAF. There is also some more preliminary evidence that dabrafenib upregulates *NEK1*,^46,47^ which given the association between disruptive *NEK1* variation and increased risk of ALS is potentially of interest.

Our genetic support from rare-variant based analyses for targets of B-vitamins, particularly amongst vitamin B_3_ and vitamin B_12_, is notable given a recent positive trial of high-dose, intravenously delivered methylcobalamin in slowing ALS progression within the first year of onset.^40^ Specifically, a phase III trial in 126 people with ALS, ultra-high doses (at 50 mg per dose) of intramuscular methylcobalamin demonstrated a 43% reduction in clinical deterioration over a 16-week treatment period, compared to placebo, with a similar incidence of adverse events in both treatment arms. This drug was subsequently approved for early stage ALS treatment in Japan; however, there have been some concerns raised about the he generalizability of the findings outside of the early periods of disease—supporting that further, longer-term trials are needed to build the evidence base for the utility of this intervention. There also are opportunities to explore other mechanisms of B_12_ supplementation, such as cyanocobalamin, which is more widely available. A trial involving nicotinamide is also ongoing (NCT04562831). The relationship between B-vitamin signalling and ALS is likely complex, and will vary between the different individual B-vitamins, as reviewed previously.^60^ Further, a previous high-throughput metabolomics Mendelian randomization-based screen on ALS liability found that a risk increasing association of leucine may arise due to decreased vitamin B_12,_^61^ with genetics likely a useful tool to further study the impacts of B_12_ biochemistry on the brain given that circulating levels of B_12_ have a strong genetic component. An adjuvant high-dose nutraceutical may be particularly clinical tractable when coupled with the existing standard-of-care, riluzole—for instance, the Oki *et al.* methylcobalamin RCT suggested that slowed functional decline was slightly stronger in those patients concomitantly treated with riluzole and methylcobalamin.^40^ The use of multiple medications, in ALS may have benefits both in the context of an orthogonal mechanism of action targeting different aspects of disease heterogeneity, as well as potential synergy. For example, both niacin and cobalamin biology have some relationship to glutamatergic excitotoxicity,^62,63^ the putative target of riluzole.

Our rare-variant based ATC code based GSEApreranked models also revealed an enrichment amongst the broad therapeutic class of corticosteroids. Glucocorticoids at time of writing have not appeared as a promising repurposing opportunity in ALS based on existing data,^64^ although there is a relative paucity of data regarding other corticosteroids like mineralocorticoids in the context of ALS. In general, aggregation of signals in a level 2 ATC code is harder to interpret, particularly as without suitable supporting pre-clinical or clinical data, this may also represent an enrichment of risk that could render a compound as deleterious for ALS. In summary, given the lack of positive pre-clinical data, we believe that corticosteroids are likely not a strong candidate but further analyses should attempt to disentangle how ALS genetic risk may influence corticosteroid signalling, which could further resolve any therapeutic tractability. This may be particularly interesting in the context of antiglucocorticoids like dazucorilant for which ALS RCTs are ongoing (NCT05407324).

Our study has a number of important limitations. First, whilst we leveraged the largest common and rare-variant association datasets available at time of analysis, more work is needed to maximize discovery power in genetic studies of ALS. There should also be particular urgency placed on increasing the representativeness of ALS genetic studies from diverse genetically defined ancestries, which should also accompany a concerted effort to make RCTs more representative, both of which with the aim of working towards equitable translation of ALS research. In general, whilst the common-variant GWAS has sufficient sample size to enable estimates of metrics including SNP heritability, the rare-variant ExWAS is still quite low powered at the sample size available for this study. As a result, we assert that replication of our rare-variant therapeutic enrichment in future larger sequencing dataset is critical to ensure that the signals detected herein are disease relevant. Further, whilst effect-sizes of common variant loci can be reasonably well estimated at current sample sizes, the common variant architecture of ALS has been found to be somewhat small compared to that of earlier onset brain disorders like schizophrenia.^65^ Moreover, the use of a ranking based approach, whilst methodologically flexible for combining inputs, also means that the enrichments uncovered herein are relative to that of all other genes, which may be less interpretable in the context of the rare-variant findings. An interesting avenue of future investigation would be to further consider more granular rare variant annotation methods as sample sizes increase—for example, *in silico* predicted loss-of-function versus gain-of-function. Our focus here, however, was not discovery at the gene-level, but to find sets of targets that are supported by multiple-lines of genomic evidence relative to other genes considered. Using our novel approach, we were able to recapitulate some previous genetic findings related to ALS repurposing that used expression-based approaches, such as some evidence for a signal in antihypertensive compounds.^14^ Through combining multiple-different methods of gene-based association, we note that we do not inherently predict whether the therapeutic mechanism of interest is beneficial or deleterious in the context of ALS. We attempted to overcome this through triangulation with existing pre-clinical and clinical data, as outlined above. Finally, the genetic association datasets used here all relate to ALS liability. An unmet need remains to also consider the relationship of putative repurposing candidates to extra-motor quality of life related measures, such as mental health challenges and pain.^66^ Given their lack of strong convergence at the gene-level we do not directly integrate common and rare-variant ranks, although we do find top-ranked genes supported by both approaches that are known as important to ALS (e.g. *NEK1*, *C9orf72*, etc.). Finally, we did not find direct genetic support for the ATC code that captures the two current FDA-approved drugs for ALS (riluzole and edaravone, N07XX)—however, given that the mechanism of action and targets of both of these drugs remain not fully resolved, further work is needed to investigate the interplay between ALS genetic risk and pathways related to these drugs like glutamatergic signalling and redox homeostasis, although our findings related to B-vitamin targets does provide some linkage to these processes.

In summary, we harnessed the complex, polygenic nature of ALS genetic liability to prioritize existing compounds and therapeutic categories that warrant additional study with respect to their utility in ALS. Specifically, we provided crucial genetic support for existing mechanisms in the ALS clinical development pipeline (RAS/RAF/MAPK and B-vitamin signalling), with novel evidence that BRAFi may be useful alone or in concert with trametinib to target the downstream, adverse neurological effects of aberrant RAS/RAF/MAPK signalling. This study also highlights the value gained by considering both common and rare-frequency components of the genetic architecture of ALS, as well as how prioritized biology may overlap with genes causative for ‘familial’ manifestations of the disorder. However, all these compounds require additional pre-clinical and clinical validation to inform their efficacy in ALS and genetic support is not sufficient to establish this. We assert that in future these candidates could be further characterized as a combination therapy with riluzole, the standard-of-care approach in many jurisdictions. Moreover, precision medicine approaches for ALS are likely to be particularly useful given its clinical heterogeneity, with a clear role for genetics in this capacity building on the success of *SOD1*-guided therapeutics.

## Supplementary Material

fcaf184_Supplementary_Data

## Data Availability

All data utilized in this study is publicly available: https://www.projectmine.com/research/download-data/. Code utilized in this study can be found at https://github.com/Williamreay/ALS_common_rare_drug_repurposing.

## References

[1] Bjelica B, Bartels MB, Hesebeck-Brinckmann J, Petri S. Non-motor symptoms in patients with amyotrophic lateral sclerosis: Current state and future directions. J Neurol. 2024;271(7):3953–3977. 38805053 10.1007/s00415-024-12455-5 PMC11233299

[2] Hardiman O, Al-Chalabi A, Chio A, et al Amyotrophic lateral sclerosis. Nat Rev Dis Primer. 2017;3:17071. 10.1038/nrdp.2017.71 28980624

[3] Feldman EL, Goutman SA, Petri S, et al Amyotrophic lateral sclerosis. Lancet Lond Engl. 2022;400(10360):1363–1380. 10.1016/S0140-6736(22)01272-7 PMC10089700 36116464

[4] van Rheenen W, van der Spek RAA, Bakker MK, et al Common and rare variant association analyses in amyotrophic lateral sclerosis identify 15 risk loci with distinct genetic architectures and neuron-specific biology. Nat Genet. 2021;53(12):1636–1648. 34873335 10.1038/s41588-021-00973-1 PMC8648564

[5] Nicolas A, Kenna KP, Renton AE, et al Genome-wide analyses identify KIF5A as a novel ALS gene. Neuron. 2018;97(6):1267–1288. 29566793 10.1016/j.neuron.2018.02.027 PMC5867896

[6] Ashburn TT, Thor KB. Drug repositioning: Identifying and developing new uses for existing drugs. Nat Rev Drug Discov. 2004;3(8):673–683. 15286734 10.1038/nrd1468

[7] Pushpakom S, Iorio F, Eyers PA, et al Drug repurposing: Progress, challenges and recommendations. Nat Rev Drug Discov. 2019;18(1):41–58. 30310233 10.1038/nrd.2018.168

[8] Reay WR, Cairns MJ. Advancing the use of genome-wide association studies for drug repurposing. Nat Rev Genet. 2021;22(10):658–671. 34302145 10.1038/s41576-021-00387-z

[9] Wang L, Lu Y, Li D, et al The landscape of the methodology in drug repurposing using human genomic data: A systematic review. Brief Bioinform. 2024;25(2):bbad527. 10.1093/bib/bbad527 PMC10818097 38279645

[10] Gerring ZF, Gamazon ER, White A, Derks EM. Integrative network-based analysis reveals gene networks and novel drug repositioning candidates for Alzheimer disease. Neurol Genet. 2021;7(5):e622. 34532569 10.1212/NXG.0000000000000622 PMC8441674

[11] Reay WR, Geaghan MP, Atkins JR, Carr VJ, Green MJ, Cairns MJ. Genetics-informed precision treatment formulation in schizophrenia and bipolar disorder. Am J Hum Genet. 2022;109(9):1620–1637. 36055211 10.1016/j.ajhg.2022.07.011 PMC9502060

[12] Woodward DJ, Thorp JG, Akosile W, et al Identification of drug repurposing candidates for the treatment of anxiety: A genetic approach. Psychiatry Res. 2023;326:115343. 37473490 10.1016/j.psychres.2023.115343 PMC10493169

[13] Reay WR, Geaghan MP, 23andMe Research Team, et al The genetic architecture of pneumonia susceptibility implicates mucin biology and a relationship with psychiatric illness. Nat Commun. 2022;13(1):3756. 35768473 10.1038/s41467-022-31473-3 PMC9243103

[14] Pain O, Jones A, Khleifat AA, et al Harnessing transcriptomic signals for amyotrophic lateral sclerosis to identify novel drugs and enhance risk prediction. Heliyon. 2023;10(15):e35342. 10.1016/j.heliyon.2024.e35342 PMC11336650 39170265

[15] Wainberg M, Sinnott-Armstrong N, Mancuso N, et al Opportunities and challenges for transcriptome-wide association studies. Nat Genet. 2019;51(4):592–599. 30926968 10.1038/s41588-019-0385-z PMC6777347

[16] Project MinE ALS Sequencing Consortium . Project MinE: Study design and pilot analyses of a large-scale whole-genome sequencing study in amyotrophic lateral sclerosis. Eur J Hum Genet. 2018;26(10):1537–1546. 29955173 10.1038/s41431-018-0177-4 PMC6138692

[17] Van Der Spek RAA, Van Rheenen W, Pulit SL, et al The project MinE databrowser: Bringing large-scale whole-genome sequencing in ALS to researchers and the public. Amyotroph Lateral Scler Front Degener. 2019;20(5–6):432–440. 10.1080/21678421.2019.1606244 PMC7893599 31280677

[18] de Leeuw CA, Mooij JM, Heskes T, Posthuma D. MAGMA: Generalized gene-set analysis of GWAS data. PLoS Comput Biol. 2015;11(4):e1004219. 25885710 10.1371/journal.pcbi.1004219 PMC4401657

[19] Li A, Liu S, Bakshi A, et al mBAT-combo: A more powerful test to detect gene-trait associations from GWAS data. Am J Hum Genet. 2023;110(1):30–43. 36608683 10.1016/j.ajhg.2022.12.006 PMC9892780

[20] Gusev A, Ko A, Shi H, et al Integrative approaches for large-scale transcriptome-wide association studies. Nat Genet. 2016;48(3):245–252. 26854917 10.1038/ng.3506 PMC4767558

[21] Zhu Z, Zhang F, Hu H, et al Integration of summary data from GWAS and eQTL studies predicts complex trait gene targets. Nat Genet. 2016;48(5):481–487. 27019110 10.1038/ng.3538

[22] Gandal MJ, Zhang P, Hadjimichael E, et al Transcriptome-wide isoform-level dysregulation in ASD, schizophrenia, and bipolar disorder. Science. 2018;362(6420):eaat8127. 10.1126/science.aat8127 PMC6443102 30545856

[23] Võsa U, Claringbould A, Westra HJ, et al Large-scale cis- and trans-eQTL analyses identify thousands of genetic loci and polygenic scores that regulate blood gene expression. Nat Genet. 2021;53(9):1300–1310. 34475573 10.1038/s41588-021-00913-z PMC8432599

[24] De Klein N, Tsai EA, Vochteloo M, et al Brain expression quantitative trait locus and network analyses reveal downstream effects and putative drivers for brain-related diseases. Nat Genet. 2023;55(3):377–388. 36823318 10.1038/s41588-023-01300-6 PMC10011140

[25] Giambartolomei C, Vukcevic D, Schadt EE, et al Bayesian test for colocalisation between pairs of genetic association studies using summary statistics. PLoS Genet. 2014;10(5):e1004383. 24830394 10.1371/journal.pgen.1004383 PMC4022491

[26] Liu Y, Xie J. Cauchy combination test: A powerful test with analytic *p* -value calculation under arbitrary dependency structures. J Am Stat Assoc. 2020;115(529):393–402. 33012899 10.1080/01621459.2018.1554485 PMC7531765

[27] Subramanian A, Tamayo P, Mootha VK, et al Gene set enrichment analysis: A knowledge-based approach for interpreting genome-wide expression profiles. Proc Natl Acad Sci U S A. 2005;102(43):15545–15550. 16199517 10.1073/pnas.0506580102 PMC1239896

[28] Wishart DS, Feunang YD, Guo AC, et al DrugBank 5.0: A major update to the DrugBank database for 2018. Nucleic Acids Res. 2018;46(D1):D1074–D1082. 29126136 10.1093/nar/gkx1037 PMC5753335

[29] Knox C, Wilson M, Klinger CM, et al DrugBank 6.0: The DrugBank knowledgebase for 2024. Nucleic Acids Res. 2024;52(D1):D1265–D1275. 37953279 10.1093/nar/gkad976 PMC10767804

[30] Lamb J, Crawford ED, Peck D, et al The connectivity map: Using gene-expression signatures to connect small molecules, genes, and disease. Science. 2006;313(5795):1929–1935. 17008526 10.1126/science.1132939

[31] Liberzon A, Birger C, Thorvaldsdóttir H, Ghandi M, Mesirov JP, Tamayo P. The molecular signatures database (MSigDB) hallmark gene set collection. Cell Syst. 2015;1(6):417–425. 26771021 10.1016/j.cels.2015.12.004 PMC4707969

[32] Fang Z, Liu X, Peltz G. GSEApy: A comprehensive package for performing gene set enrichment analysis in python. Bioinformatics. 2023;39(1):btac757. 36426870 10.1093/bioinformatics/btac757 PMC9805564

[33] Ziff OJ, Neeves J, Mitchell J, et al Integrated transcriptome landscape of ALS identifies genome instability linked to TDP-43 pathology. Nat Commun. 2023;14(1):2176. 37080969 10.1038/s41467-023-37630-6 PMC10119258

[34] Cheng F, Li W, Zhou Y, et al admetSAR: A comprehensive source and free tool for assessment of chemical ADMET properties. J Chem Inf Model. 2012;52(11):3099–3105. 23092397 10.1021/ci300367a

[35] DeJesus-Hernandez M, Mackenzie IR, Boeve BF, et al Expanded GGGGCC hexanucleotide repeat in noncoding region of C9ORF72 causes chromosome 9p-linked FTD and ALS. Neuron. 2011;72(2):245–256. 21944778 10.1016/j.neuron.2011.09.011 PMC3202986

[36] Iacoangeli A, Fogh I, Selvackadunco S, et al SCFD1 expression quantitative trait loci in amyotrophic lateral sclerosis are differentially expressed. Brain Commun. 2021;3(4):fcab236. 10.1093/braincomms/fcab236 PMC8545614 34708205

[37] Li CY, Yang TM, Ou RW, Wei QQ, Shang HF. Genome-wide genetic links between amyotrophic lateral sclerosis and autoimmune diseases. BMC Med. 2021;19(1):27. 33541344 10.1186/s12916-021-01903-y PMC7863260

[38] Mann JR, McKenna ED, Mawrie D, et al Loss of function of the ALS-associated NEK1 kinase disrupts microtubule homeostasis and nuclear import. Sci Adv. 2023;9(33):eadi5548. 10.1126/sciadv.adi5548 PMC10431718 37585529

[39] Kelleher KJ, Sheils TK, Mathias SL, et al Pharos 2023: An integrated resource for the understudied human proteome. Nucleic Acids Res. 2023;51(D1):D1405–D1416. 36624666 10.1093/nar/gkac1033 PMC9825581

[40] Oki R, Izumi Y, Fujita K, et al Efficacy and safety of ultrahigh-dose methylcobalamin in early-stage amyotrophic lateral sclerosis: A randomized clinical trial. JAMA Neurol. 2022;79(6):575–583. 35532908 10.1001/jamaneurol.2022.0901 PMC9086935

[41] Hanna M, Jaqua E, Nguyen V, Clay J. B vitamins: Functions and uses in medicine. Perm J. 2022;26(2):89–97. 35933667 10.7812/TPP/21.204 PMC9662251

[42] Torres P, Cabral-Miranda F, Gonzalez-Teuber V, Hetz C. Proteostasis deregulation as a driver of C9ORF72 pathogenesis. J Neurochem. 2021;159(6):941–957. 34679204 10.1111/jnc.15529

[43] Lipinski CA . Lead- and drug-like compounds: The rule-of-five revolution. Drug Discov Today Technol. 2004;1(4):337–341. 24981612 10.1016/j.ddtec.2004.11.007

[44] Gouda MA, Subbiah V. Precision oncology for BRAF-mutant cancers with BRAF and MEK inhibitors: From melanoma to tissue-agnostic therapy. ESMO Open. 2023;8(2):100788. 36842301 10.1016/j.esmoop.2023.100788 PMC9984800

[45] Yao Z, Gao Y, Su W, et al RAF inhibitor PLX8394 selectively disrupts BRAF dimers and RAS-independent BRAF-mutant-driven signaling. Nat Med. 2019;25(2):284–291. 30559419 10.1038/s41591-018-0274-5 PMC6404779

[46] Klaeger S, Heinzlmeir S, Wilhelm M, et al The target landscape of clinical kinase drugs. Science. 2017;358(6367):eaan4368. 10.1126/science.aan4368 PMC6542668 29191878

[47] Bromberger S, Zadorozhna Y, Ressler JM, et al Off-targets of BRAF inhibitors disrupt endothelial signaling and vascular barrier function. Life Sci Alliance. 2024;7(8):e202402671. 38839106 10.26508/lsa.202402671 PMC11153892

[48] Mead RJ, Shan N, Reiser HJ, Marshall F, Shaw PJ. Amyotrophic lateral sclerosis: A neurodegenerative disorder poised for successful therapeutic translation. Nat Rev Drug Discov. 2023;22(3):185–212. 36543887 10.1038/s41573-022-00612-2 PMC9768794

[49] Diab R, Pilotto F, Saxena S. Autophagy and neurodegeneration: Unraveling the role of C9ORF72 in the regulation of autophagy and its relationship to ALS-FTD pathology. Front Cell Neurosci. 2023;17:1086895. 37006471 10.3389/fncel.2023.1086895 PMC10060823

[50] Webster CP, Smith EF, Bauer CS, et al The C9orf72 protein interacts with Rab1a and the ULK1 complex to regulate initiation of autophagy. EMBO J. 2016;35(15):1656–1676. 27334615 10.15252/embj.201694401 PMC4969571

[51] Settembre C, Di Malta C, Polito VA, et al TFEB links autophagy to lysosomal biogenesis. Science. 2011;332(6036):1429–1433. 21617040 10.1126/science.1204592 PMC3638014

[52] Li S, Song Y, Quach C, et al Transcriptional regulation of autophagy-lysosomal function in BRAF-driven melanoma progression and chemoresistance. Nat Commun. 2019;10(1):1693. 30979895 10.1038/s41467-019-09634-8 PMC6461621

[53] Foth M, McMahon M. Autophagy inhibition in BRAF-driven cancers. Cancers (Basel). 2021;13(14):3498. 34298710 10.3390/cancers13143498 PMC8306561

[54] Chun YS, Kim MY, Lee SY, et al MEK1/2 inhibition rescues neurodegeneration by TFEB-mediated activation of autophagic lysosomal function in a model of Alzheimer’s disease. Mol Psychiatry. 2022;27(11):4770–4780. 35948663 10.1038/s41380-022-01713-5 PMC9734062

[55] Caldi Gomes L, Hänzelmann S, Hausmann F, et al Multiomic ALS signatures highlight subclusters and sex differences suggesting the MAPK pathway as therapeutic target. Nat Commun. 2024;15(1):4893. 38849340 10.1038/s41467-024-49196-y PMC11161513

[56] Goz RU, Akgül G, LoTurco JJ. BRAFV600E expression in neural progenitors results in a hyperexcitable phenotype in neocortical pyramidal neurons. J Neurophysiol. 2020;123(6):2449–2464. 32401131 10.1152/jn.00523.2019 PMC7311733

[57] Ye Q, Srivastava P, Al-Kuwari N, Chen X. Oncogenic BRAFV600E induces microglial proliferation through extracellular signal-regulated kinase and neuronal death through c-jun N-terminal kinase. Neural Regen Res. 2023;18(7):1613–1622. 36571370 10.4103/1673-5374.361516 PMC10075110

[58] Mass E, Jacome-Galarza CE, Blank T, et al A somatic mutation in erythro-myeloid progenitors causes neurodegenerative disease. Nature. 2017;549(7672):389–393. 28854169 10.1038/nature23672 PMC6047345

[59] King AJ, Arnone MR, Bleam MR, et al Dabrafenib; preclinical characterization, increased efficacy when combined with trametinib, while BRAF/MEK tool combination reduced skin lesions. PLoS One. 2013;8(7):e67583. 23844038 10.1371/journal.pone.0067583 PMC3701070

[60] Goncharova PS, Davydova TK, Popova TE, et al Nutrient effects on motor neurons and the risk of amyotrophic lateral sclerosis. Nutrients. 2021;13(11):3804. 34836059 10.3390/nu13113804 PMC8622539

[61] Boddy S, Islam M, Moll T, et al Unbiased metabolome screen leads to personalized medicine strategy for amyotrophic lateral sclerosis. Brain Commun. 2022;4(2):fcac069. 35441136 10.1093/braincomms/fcac069 PMC9010771

[62] Akaike A, Tamura Y, Sato Y, Yokota T. Protective effects of a vitamin B12 analog, methylcobalamin, against glutamate cytotoxicity in cultured cortical neurons. Eur J Pharmacol. 1993;241(1):1–6. 7901032 10.1016/0014-2999(93)90925-8

[63] Obrador E, Salvador-Palmer R, López-Blanch R, Dellinger RW, Estrela JM. NAD+ precursors and antioxidants for the treatment of amyotrophic lateral sclerosis. Biomedicines. 2021;9(8):1000. 34440204 10.3390/biomedicines9081000 PMC8394119

[64] Goslinga JA, Terrelonge M, Bedlack R, et al ALSUntangled #65: Glucocorticoid corticosteroids. Amyotroph Lateral Scler Front Degener. 2023;24(3–4):351–357. 10.1080/21678421.2022.2099746 35997522

[65] Schizophrenia Working Group of the Psychiatric Genomics Consortium, Ripke S, Walters JT, O’Donovan MC. Mapping genomic loci prioritises genes and implicates synaptic biology in schizophrenia. Genetic and Genomic Medicine; 2020.

[66] Martinez H, Moreno-Cuevas J, Gonzalez-Garza MT, et al Extra-motor symptoms in amyotrophic lateral sclerosis. Analysis of 112 patients (P7.034). Neurology. 2014;82(10_supplement):P7.034.

