# Supplementary material for: Drug repurposing candidates for amyotrophic lateral sclerosis using common and rare genetic variants
Source: Brain Commun. 2025 May 9;7(3):fcaf184. doi: 10.1093/braincomms/fcaf184 (PMC12089939; doi:10.1093/braincomms/fcaf184)
Supplement: fcaf184_Supplementary_Data [file fcaf184_supplementary_data.zip › Supplementary_Materials_and_Figures.pdf]

Drug repurposing candidates for amyotrophic lateral sclerosis using common and rare genetic variants

Supplementary Materials

SUPPLEMENTARY MATERIALS AND METHODS 2

SUPPLEMENTARY RESULTS 7

SUPPLEMENTARY FIGURES 9

SUPPLEMENTARY REFERENCES 23

SUPPLEMENTARY MATERIALS AND METHODS

Description of common variant based ranking approaches

We describe forthwith each of the gene-based methods used in this study in some additional detail.

MAGMA

MAGMA (Multi-marker Analysis of GenoMic Annotation) is a gene-based association method that assigns variants to genes based on their physical position relative to that of genic coordinates[1]. Here, we leveraged genic coordinates outside of the major histocompatibility complex region (MHC) on chromosome six sourced from the MAGMA website (https://cncr.nl/research/magma/), with genic coordinates extended 5 kilobases (kb) upstream and 1.5 kb downstream to capture regulatory variation. After mapping variants to genes, variant-wise *P*-values were transformed to a *Z*-value through leveraging the probit function. The test-statistic used for MAGMA is the sum of squared *Z*-statistics in a gene, with the distributional assumption for the vector of Z that $Z \sim \mathcal{N}(0, \Sigma)$, where Σ denotes a correlation matrix of genotypes derived using estimates of linkage disequilibrium (LD) from the 1000 genomes phase III European reference panel. Gene-based *P*-values can then be calculated from this test-statistic using an integration based approach proposed by Imhof that directly evaluates the sampling distribution. As a result, gene-wise *P* values can be estimated that account for LD between variants.

mBAT-combo

We also used an alternate physical position based method for comparison with MAGMA termed the multivariate set-based association test (mBAT) – specifically, we implemented a hybrid method (mBAT-combo) that meta-analyses the mBAT gene-based *P* value with that of a related gene-based method (fastBAT) through leveraging the properties which arise from the heavy tail of the Cauchy distribution to account for *P*-value dependency[2,3]. A key difference between mBAT and MAGMA is that it mBAT purportedly optimised for the scenario of 'masking effects', which arises when there is negative covariance amongst causal variants at a locus. The mBAT method leverages the quadratic form theory in normal variables to test for non-zero variance explained by variants in a set, here a gene. This differs from approaches like MAGMA and fastBAT which compute the sum of χ^2 statistics – for example, in MAGMA the test statistic is equal to a mixture distribution of χ^2 statistics with one degree of freedom. However, as the improved performance of the mBAT approach over a summed χ^2 approach may vary with different local and global genetic architectures, the mBAT-combo combines gene-wise *P*-values from mBAT with fastBAT via the Cauchy combination method. This involves transforming the two input *P* values to approximate Cauchy distribution, which due to its heavy tail, is insensitive to covariance between the input *P*-values that arise from their dependency (calculated on same GWAS). Analogous to MAGMA, this method results in the estimation of a gene-wise *P*-value that can be compared to other methods for ranking.

TWAS

The transcriptome-wide association study (TWAS) method for gene-based association differs from both MAGMA and mBAT-combo in that it relies on the association between variants and mRNA expression to assign variant effects to a gene, as reviewed extensively elsewhere. In this study, we used the FUSION method for TWAS[4]. Briefly, FUSION uses pre-trained multivariate models of genetically regulated expression (GReX) which seek to optimise the variance explained in gene-wise mRNA expression using *cis*-acting genetic variants through comparing competing models, such as penalised regression approaches (i.e., elastic net and LASSO) and Bayesian sparse linear mixed models. These GReX models are only retained for further analysis in FUSION if the directly estimated heritability by GREML using only *cis*-acting factors is significantly non-zero. Here, we utilised pre-trained GReX model weights from post-mortem cortical samples from the PsychENCODE consortium that underwent SNP genotyping and RNA sequencing, as outlined previously[5]. The TWAS test statistic leverages the transpose matrix of variant weights from the GReX model for a gene (ω'_{GReX}), adjusted for LD, with that of the marginal variant-wise association of those same variants with ALS liability (Z_{ALS}) such that $Z_{TWAS} = {\omega'_{GReX} Z_{ALS}} / {\mathrm{var}(\omega'_{GReX} Z_{ALS})}$. In the case of $Z_{TWAS} > 0$, this implies that increased genetically predicted expression of the gene is associated with increased ALS liability, and *vice versa* for a negative sign test-statistic.

SMR

We also used an alternate expression based approach termed summary-data based Mendelian randomisation (SMR)[6]. Rather than leveraging variant weights from a multivariate GReX model, SMR utilises specific variants strongly associated with mRNA expression as instrumental variables (IVs). The SMR effect size denotes the potential causal effect of genetically predicted expression of a gene on ALS liability by using the association of an effect allele with the log odds of ALS divided by its corresponding association with gene expression. The standard error is approximated using Delta method. We used precalculated input IVs for SMR from two tissues given the large number of genes available for both – brain (MetaBrain) and whole blood (eQTLgen). This was done to capture both central nervous system and systemic elements of ALS liability that may be mediated by mRNA expression, as well the fact that using both tissues to source IVs resulted in a suitable number of gene-wise results available for ranking.

Colocalisation adjusted TWAS

A limitation of TWAS is that a significantly non-zero TWAS Z in either direction does not imply a causal relationship between genetically predicted expression and ALS liability due to the influence of factors such as co-regulation and confounding arising from linkage-based effects[4]. One method proposed to further interrogate marginal TWAS associations are Bayesian colocalisation tests, such as the *coloc* framework[7]. Briefly, this approach tests competing five prior hypotheses ($H_{0\text{-}4}$) related to the association of a set of variants in a region between two traits. These are as follows: H_0 = there is no association of the region with either trait, H_1 = the region is associated with trait one only, H_2 = the region is associated with trait two only, H_3 = the region is associated with both traits but is driven by a different underlying causal variant,

and H_4 = the region is associated with both traits but is driven by the same underlying causal variant. Given a collection of priors placed on each hypothesis, the marginal GReX weights and their corresponding ALS liability association are used to integrate over the entire hypothesis space to estimate posterior probabilities (*PP*) of each hypothesis. Here, we are interested in the *PP* of H_4, which suggests a direct relationship between the GReX signal and that of ALS, although we note that the assumption of a single causal variant may perform poorly in the complex regions driven by multiple-causal variants or even common loci that tag tandem repeat-based signal, as described in the manuscript for *C9orf72*. Nonetheless, we ranked the product of the TWAS *Z* and PP_{H4} as an alternative rank input to that of the marginal TWAS *Z* as it provides a competing metric of confidence that can be incorporated into our common-variant led framework such that genes with evidence of a shared causal variant (under this single causal variant assumption) are upweighted. Adaption of colocalisation to the scenario of multiple-causal variants remains challenging without access to in-sample LD matrices and the single causal variant approach remains computationally efficient and scalable; however, in future, this approach could be adapted such that the priors used are reflective of a multiple-causal variant configuration.

HEIDI adjusted SMR

Somewhat analogous to the above, SMR also may be biased in the presence of factors which give rise to spurious associations between genetically predicted expression, as indexed by strongly associated eQTLs as IVs, and ALS liability. Here, we use an alternate frequentist-based approach termed HEIDI (heterogeneity in dependent instruments) to assess whether the expression and ALS signal is driven by the same causal variant. HEIDI tests the null-hypothesis of the association arising due to linkage, and a result, significant *P*-values below an *a priori* specified alpha can be considered as the threshold for genes of which the association between expression and ALS may be driven by confounding linkage. We leveraged this approach with our SMR results by considering all genes with at least a nominal HEIDI *P*-value ($P < 0.05$), which is very conservative, although useful in this context given the well-powered nature of the marginal SMR test-statistics. Relative to the Bayesian colocalisation approach, a *P*-value has a quite different interpretation from posterior probabilities which are interpretable between 0% and 100%. As a result, we use the HEIDI *P*-value for scaling as follows – firstly, the HEIDI *P*-value is converted to a *Z* score, followed by dividing the marginal SMR *Z* score by the HEIDI *Z* score only in the case of HEIDI $|Z| < 1.96$, indicative of nominal statistical significance. This down-weights genes with evidence of a signal driven by linkage but leaves those without such evidence unaltered, although the with the caveats of assuming a single causal variant, as described above.

Variant masks used in van Rheenen *et al.* ExWAS

As outlined in the main text, the ExWAS performed by van Rheenen *et al.* used a series of variant masks at the minor allele frequency (MAF) thresholds of 0.01, and 0.005, respectively[8]. We describe these forthwith for completeness. Disruptive variation was defined in that study as those variants annotated by the SnpEff annotation package as one of the following: frameshift, splice site, exon loss, stop gained, start loss or transcription ablation. Damaging loci were missense variants predicted as damaging by seven *in silico* methods SIFT, PolyPhen-2,

LRT, MutationTaster-2, Mutations Assessor, and PROVEAN. All other missense ('non-damaging' missense) were those for which the aforementioned seven approaches did not uniformly predict deleteriousness, although this does not preclude that some of those loci may in fact be damaging given the inherent strengths and limitations of each approach. We note that for this study we had to use these pre-computed variant masks as only transcript-level association results were made publicly available.

Combining rare-variant transcript level burden testing into a gene-based metric

In the ALS GWAS/ExWAS paper from which the ALS data for this study is sourced, the ExWAS burden testing was conducted at the level of individual transcripts with at least five individuals with a non-zero burden of the variant mask tested, rather than that of genes. We wished here to combine these transcript-level *P* values from the burden testing analyses into a consolidated gene-based *P*-values – however, many existing approaches for combining *P*-values either assume independence (e.g., Fisher's method) or require a metric of covariation between *P*-values if this independence assumption is violated (e.g., Brown's method). Instead, we chose to implement a more recent approach for *P*-value meta-analysis which is largely immune to inflation that arises from covariance between *P*-values as these *P*-values arise from the same sample (Cauchy combination). This approach leverages the behaviour of the Cauchy distribution as follows. The test statistic (*T*) is a sum of P-values (P_i) transformed to approximate a Cauchy distribution $T = \tan\{(0.5 - P_i)\pi$. *T* is insensitive to correlations amongst the *P* values that arise due to these *P* values being from the same sample, due to the heavy tail of the Cauchy distribution, as described previously[3]. The combined, gene-level meta-analytic *P* value (P_{Gene}) was approximated using the cumulative density function of the Cauchy distribution as follows:

$$P_{\text{Gene}} = 1/2 - \left[\arctan(T/w) / \pi \right].$$

***NEK1* related analyses**

As *NEK1* has been supported as an ALS risk gene through both common and rare variant approaches, we conducted additional analyses to interrogate its relationship to ALS genetic liability. Firstly, we identified gene and phenotype ontology gene-sets curated by g:Profiler of which *NEK1* is a member[9]. The association of these gene-sets with ALS was then tested based on the MAGMA gene-set association approach which specifically evaluates the enrichment of the ALS polygenic common-variant signal in each set relative to all remaining genes, as described elsewhere[1]. These gene-set association analyses were conducted using gene-level association *P*-values from MAGMA whereby SNPs were assigned to genes using a conservative extension of genic boundaries to capture regulatory effects, as described above (5kb upstream, 1.5 kb downstream), as well as a liberal genic boundary for comparative purposes (35 kb upstream, 10 kb downstream). Secondly, we conducted a phenome-wide Mendelian randomisation study (MR-pheWAS) using a *NEK1* brain eQTL as an instrumental variable for CNS *NEK1* expression, in line with our previous work applying this MR-pheWAS approach[10,11]. Briefly, this involved selecting a brain *NEK1* eQTL from the eQTL catalogue

resource with strong support from genetic finemapping (rs10520157, GTEx cerebellum, posterior inclusion probability in credible set = 86%). The effect of genetically predicted brain *NEK1* expression was then estimated on every clinical endpoint in FinnGen release 10 with at least 1000 cases using the Wald ratio method ($N_{Endpoints}$ = 1207). Multiple-testing correction was performed using the Bonferroni method to account for 1207 tests ($P < 4.14 \times 10^{-5}$). We then sought to replicate the findings with respect to the top association signals using GWAS assembled by the database IEUGWASdb v.8.2.3 via its interrogation with the ieugwasr package v0.1.5. In all cases, we flipped the sign of the MR beta estimate to represent effect sizes per standard deviation *decrease* in genetically predicted *NEK1* expression.

SUPPLEMENTARY RESULTS

Summary characteristics of common and rare variant led ranks

We first implemented a novel pipeline that uses different approaches to estimate gene-level associations with ALS for common and rare variation, separately. In terms of common variant ranks (N_{Genes} = 19,888), the modal number of ranks available for a gene of eight ranks was six (Supplementary Figure 1, Supplementary Table 2). For genes with at least five non-missing ranks (N_{Genes} = 8,764), the median coefficient of variation (CV) between ranks was 59.35% [interquartile range (IQR) = 33.68%, Supplementary Figure 2]. The correlations estimated between common variant ranking approaches were all positive, moderate-to-large [$\rho > 0.25$, Spearman's rho (ρ)], and statistically significant ($P < 0.01$, Supplementary Text, Supplementary Figure 3). The unadjusted expression-based annotation ranks (TWAS-Brain, SMR-Brain, SMR-Blood) all exhibited large correlations ($\rho > 0.8$) with their adjusted ranks (colocalisation and HEIDI adjusted, respectively) – for example, the correlation between TWAS *Z* and the colocalisation adjusted TWAS *Z* was = 0.88 (Supplementary Figure 3). The modal number of ranks available for rare variants (N_{Genes} = 17,489) was also six out of a maximum of eight, with the variability between ranks for the same genes that had at least five input ranks (N_{Genes} = 9,365) analogous to that of the common variant approach [median CV = 58.5% (IQR = 43.73%), Supplementary Figure 4, Supplementary Table 3]. Correlations were estimated between non-synonymous ranks, as well as with synonymous variants as a positive control (Supplementary Figure 5). This revealed that synonymous gene-based ranks were not correlated with those using disruptive or damaging variant annotations at either MAF < 0.01 or MAF < 0.005. Notably, disruptive and damaging ranks were not significantly correlated, suggesting these two variant types point to different aspects of risk for ALS, although at current ExWAS sample sizes this is somewhat speculative

NEK1 gene-set association and phenome-wide Mendelian randomisation

Using a conservative gene-set association approach (MAGMA, competitive test), we tested common variant enrichment amongst 703 biological and phenotype ontology sets of which *NEK1* was a member (Supplementary Figure 7). Whilst no sets survived Bonferroni correction, we observed nominally significant enrichments in ALS relevant gene-sets such as *Laryngospasm* ($P_{\text{MAGMA-GSA}} = 1.57\text{x}10^{-4}$, liberal genic boundaries) and *Motor neuron atrophy* ($P_{\text{MAGMA-GSA}} = 4.23\text{x}10^{-4}$, conservative genic boundaries). This suggests that *NEK1* is related to pathologies for which the wider ALS common polygenic architecture is enriched based on positional mapping of variants to genes, although larger ALS GWAS are still needed to better estimate these enrichments. Finally, we sought to evaluate the association between the ALS risk increasing direction of *NEK1* expression/function (decreased) and clinical endpoints across the human clinical phenome in the independent FinnGen study (Supplementary Table 4, Supplementary Figure 7). Whilst no *NEK1* associations surpassed strict Bonferroni correction, we found a supportive nominal association between decreased genetically predicted *NEK1* brain expression and increased odds of two endpoints: 'soft tissue disorders' ($P = 5.1 \text{ x } 10^{-4}$) and 'pain in limb' ($P = 1.1 \text{ x } 10^{-3}$). The odds increasing relationship between decreased genetically predicted *NEK1* brain expression and peripheral pain was nominally replicated

using a UK Biobank GWAS - ‘self reported hip pain experienced in the last month’ ($P = 5.7 \times 10^{-5}$).

SUPPLEMENTARY FIGURES

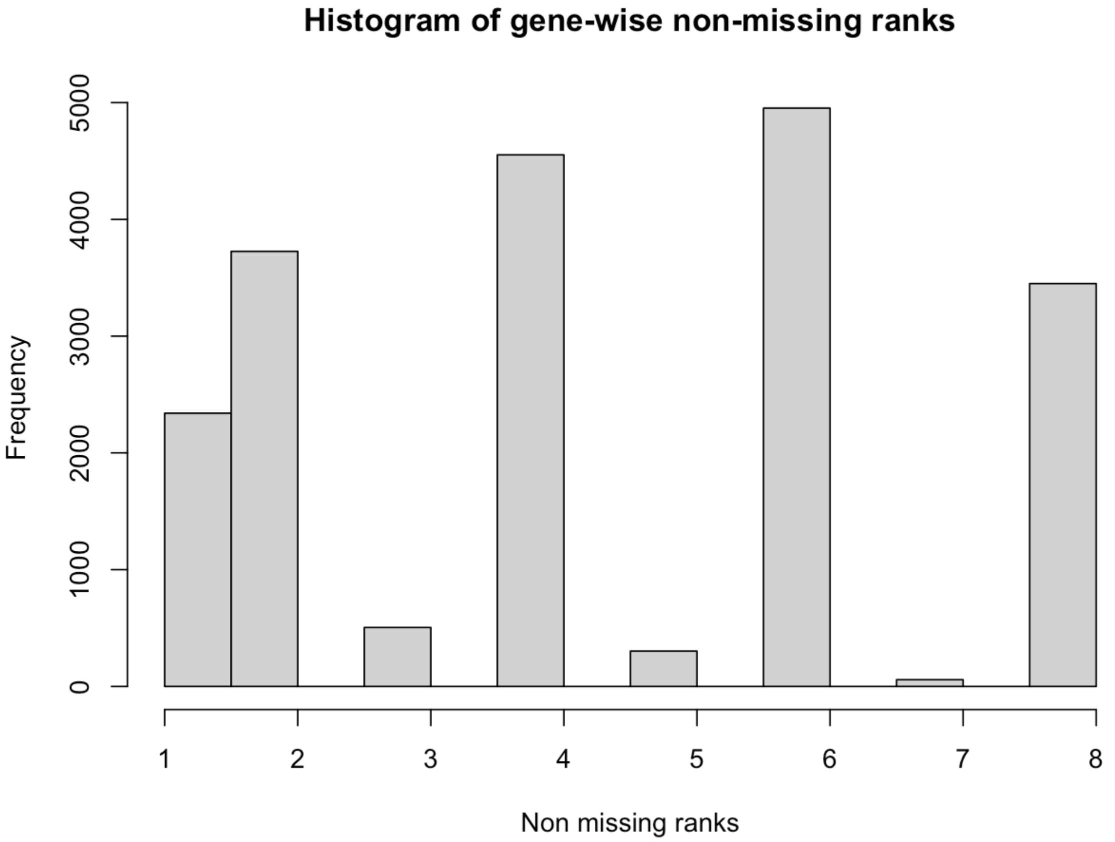

Supplementary Figure 1. Histogram of the counts of non-missing ranks per gene using the common variant-led approach. Only genes with at least one rank were considered (N_{Genes} = 19,888).

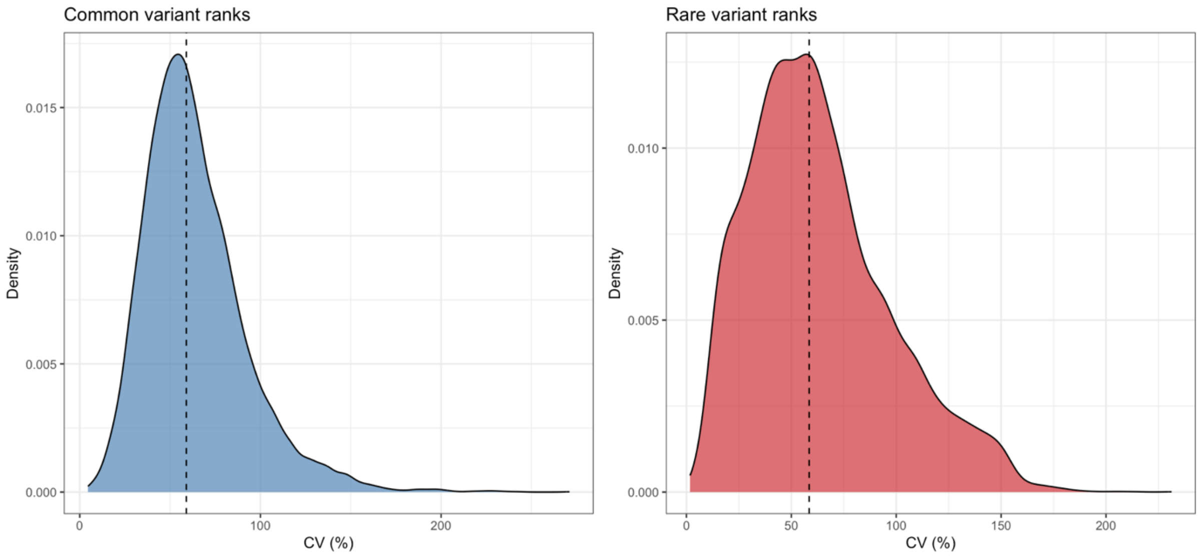

Supplementary Figure 2. The distribution of coefficient of variation estimates between ranks. A coefficient of variation was calculated through comparing gene-wise standard deviation of ranks, to that of the mean rank, and converted to a percentage by multiplying by 100 (CV%). The left-hand plot denotes the smoothed density of CV% for the common variant-led approach with sufficient ranks to calculate CV (N_{Genes} = 8764), whilst the right-hand plot visualises the CV% for the rare variant-led approach N_{Genes} = 9365).

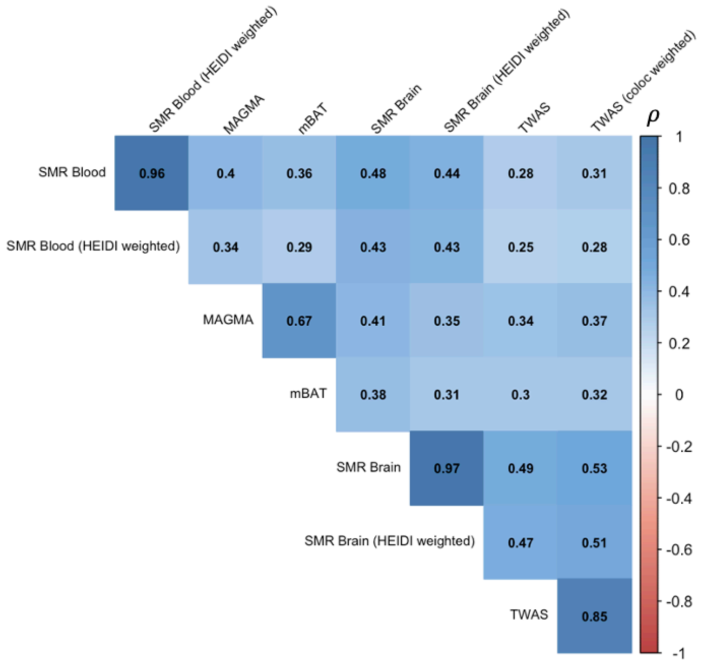

Supplementary Figure 3. Correlations between the input ranks for each common variant-led approach. All correlation (Spearman's rho) estimates denoted, with larger estimates shaded a darker blue – total number of genes ranked using common variant approach = 19,888.

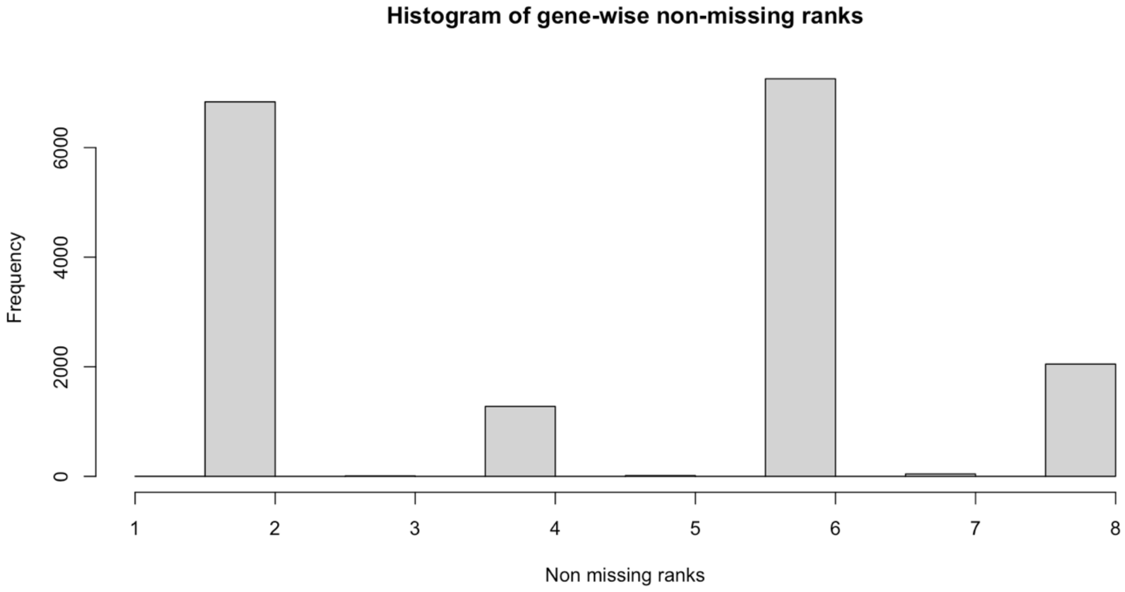

Supplementary Figure 4. Histogram of the counts of non-missing ranks per gene using the rare variant-led approach. Only genes with at least one rank were considered (N_{Genes} = 17,489).

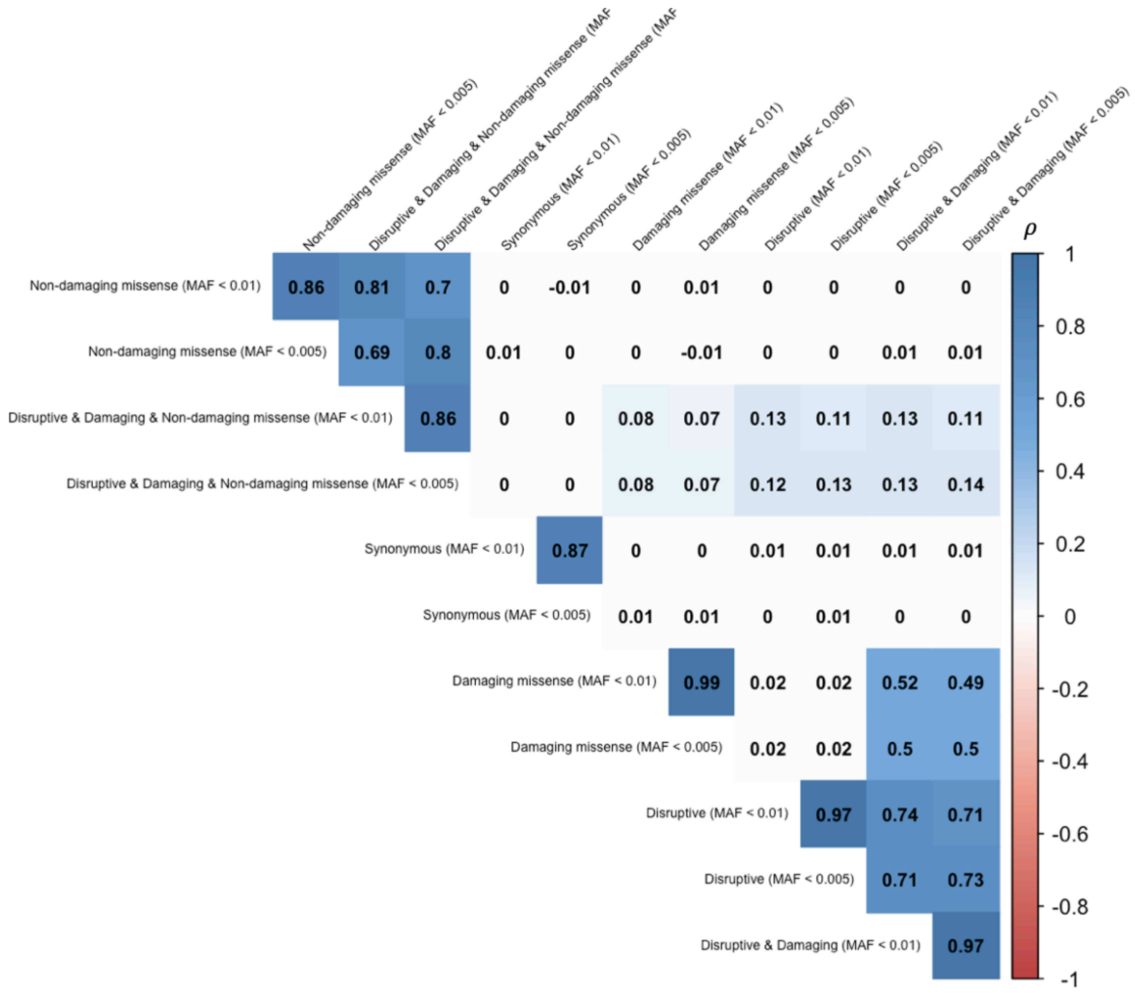

Supplementary Figure 5. Correlations between the input ranks for each rare variant-led approach. All correlation (Spearman's rho) estimates denoted, with larger estimates shaded a darker blue (N_{Genes} = 17,489).

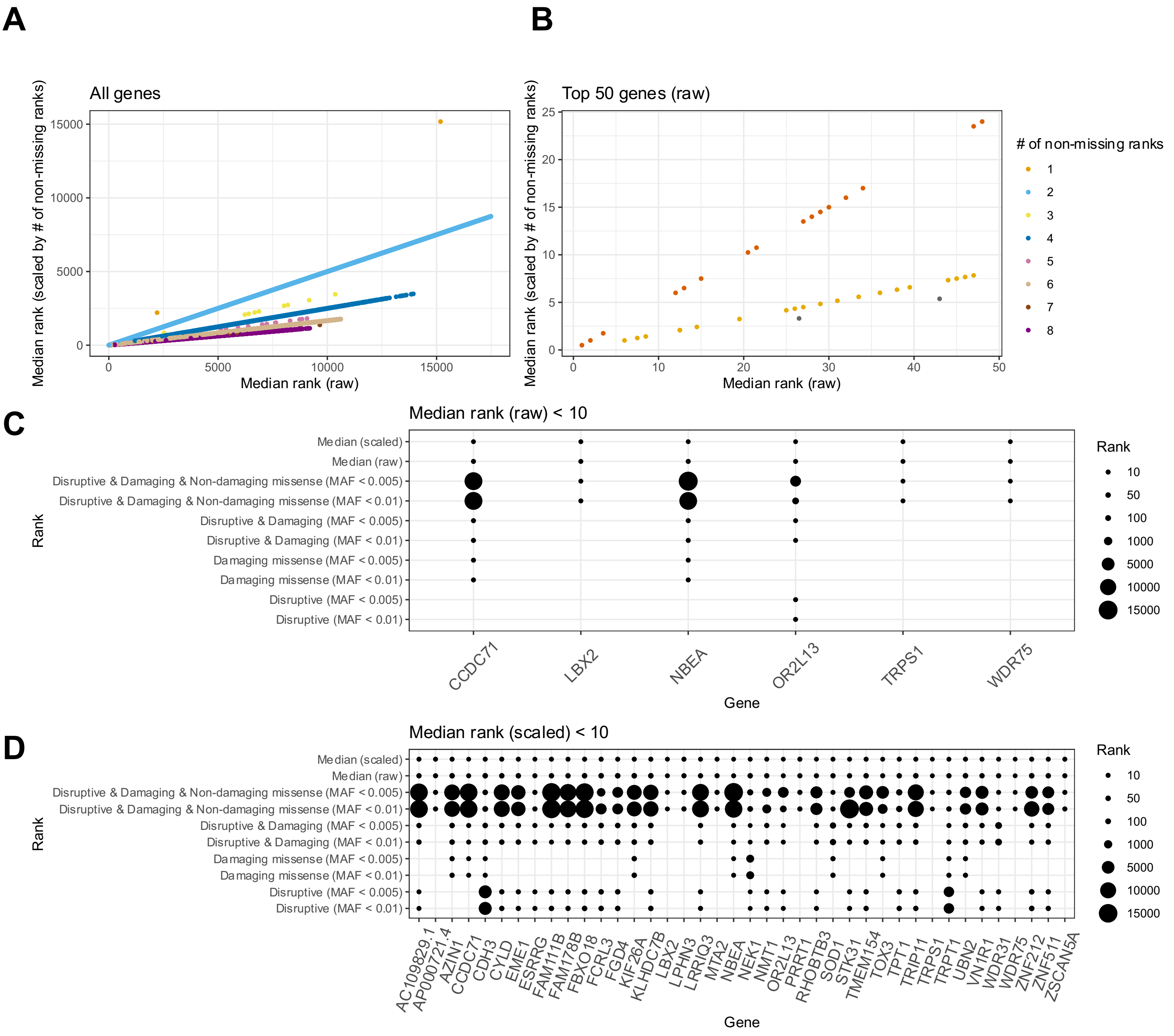

Supplementary Figure 6. Rare-variant led ranking of genes using amyotrophic lateral sclerosis (ALS) exome-wide association study (ExWAS) data. In this approach, eight different gene-based methods are leveraged to rank genes in an ascending fashion, such that the top gene is ranked '1'. Each point denotes rare variant ranks derived from ExWAS with sample size of 6,538 ALS cases, 2,415 controls). In panel (**a**) the x-axis denotes the median rank across all gene-wise input ranks, whilst the y-axis denotes the scaled median rank that upweights genes with a greater number of non-missing input ranks. This scatter plot visualises the relationship between the raw and scaled median rank, with points coloured by the number of non-missing ranks per-gene (no statistical comparison performed between raw and scaled metrics). The same relationship is plotted in panel (**b**) with each point denoting just the genes ranked in the top 50 using the raw median. (**c**) Genes with raw median rank < 10 are visualised in terms of their input rank for each method, with each point representing the rank for that

method and whitespace denoting a rank not available for that method. (**d**) Analogous to panel c, but genes with a scaled median rank < 10 are visualised.

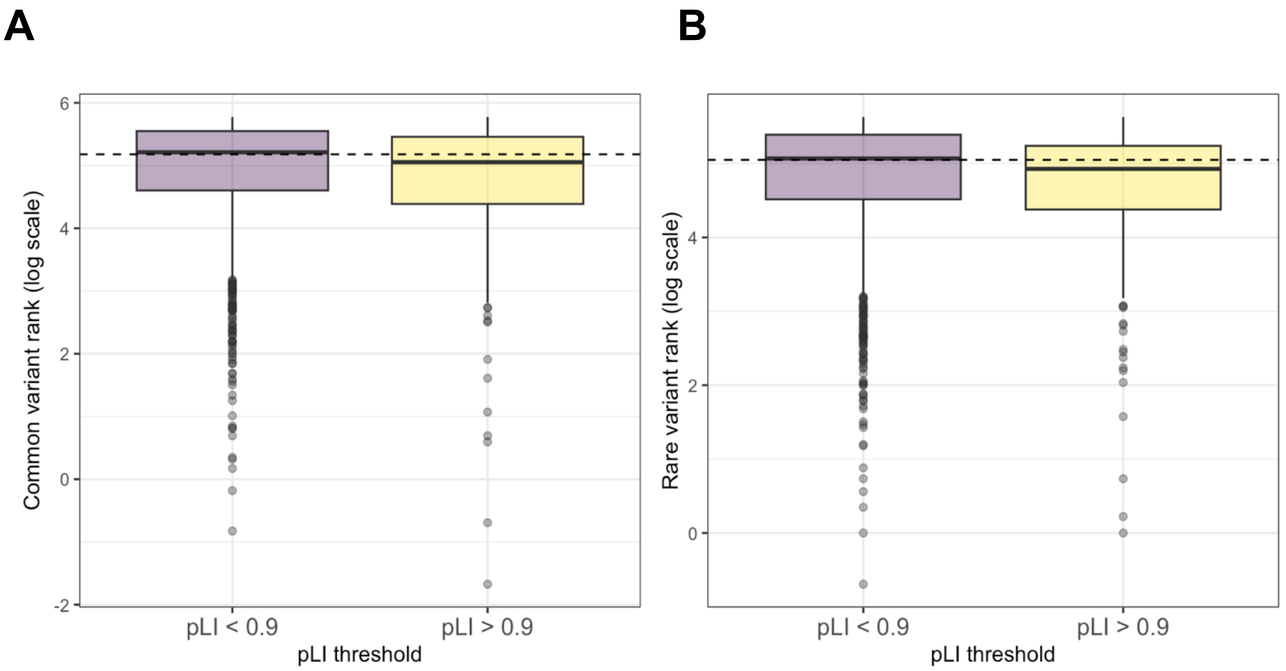

Supplementary Figure 7. The distribution of gene-based ranks in the 10% closest to one relative to the predicted probability of loss-of-function intolerance. Each point represents the natural log transformed median scaled rank for a gene, with best ranked genes in the 10% closes to one plotted. The gene-based ranks visualised for genes with a probability of loss of function intolerance (pLI) greater than 0.9 (90%) relative to all other genes. (**a**) Common variant ranks, (**b**) Rare variant ranks There was a significant difference between the two groups for both common and rare variant genes – common variant: $P = 0.022$ ($W = 210030$, Wilcoxon rank sum test with continuity correction, $N_{Genes} = 1740$); rare variant: $P = 7.04 \times 10^{-4}$ ($W = 194654$, Wilcoxon rank sum test with continuity correction, $N_{Genes} = 1727$).

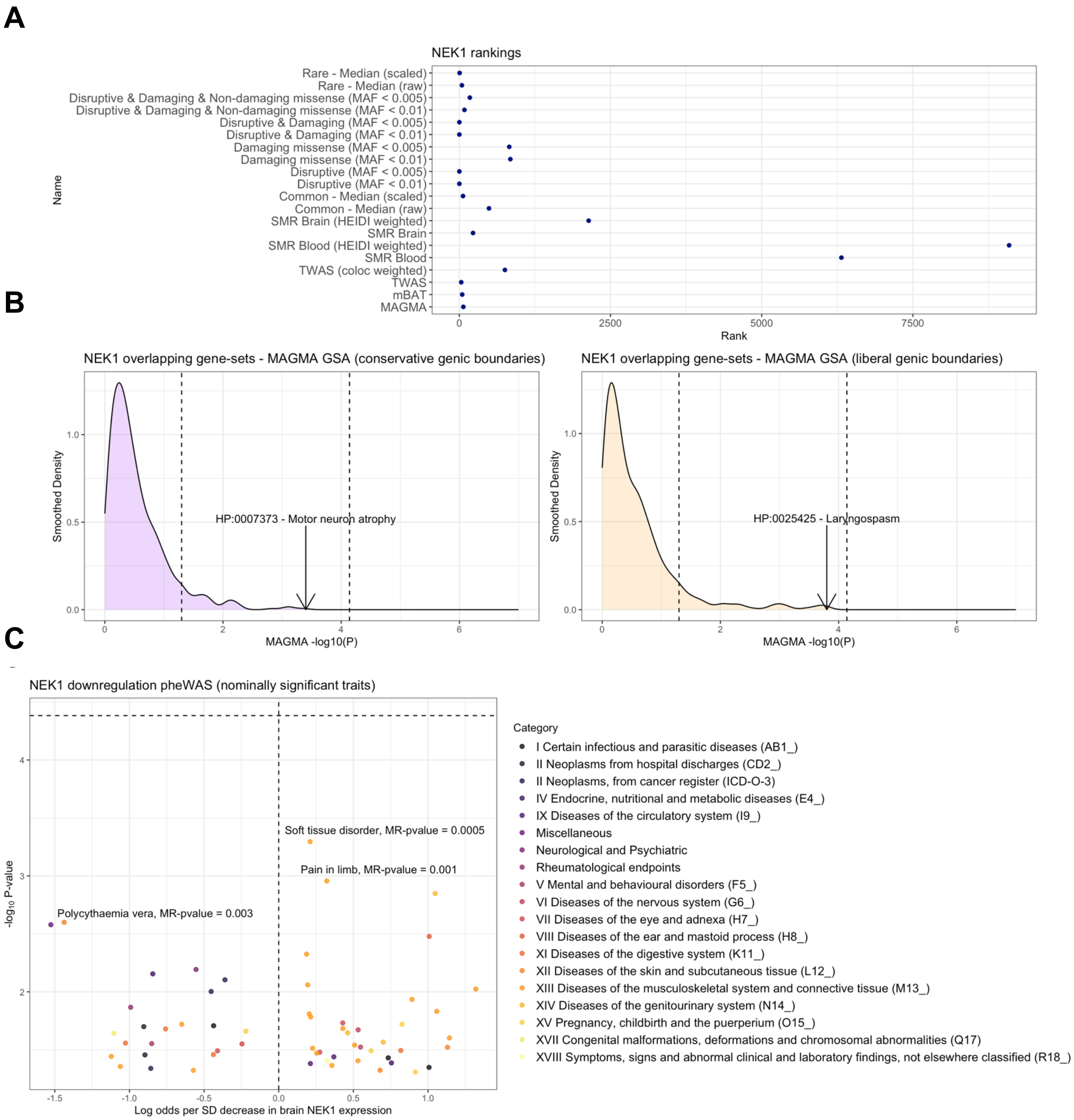

Supplementary Figure 8. Exploring the role of *NEK1* genetic variation in liability to amyotrophic lateral sclerosis (ALS). (**a**) Input ranks across the common and rare variant-led approaches for *NEK1* - rare variant ranks derived from exome-wide association study (ExWAS) with sample size of 6,538 ALS cases, 2,415 controls), whilst common-variant ranks were derived from genome-wide association study

(27,205 ALS cases and 110,881 controls. (**b**) MAGMA gene-set association (GSA) using ALS common variant risk amongst ontological pathways of which *NEK1* is a member. The smoothed density of $-\log_{10}$ transformed *P*-values GSA are plotted, with a dotted line representing both nominal significance and Bonferroni significance. The top pathway is highlighted. The left-panel denotes MAGMA GSA method using conservative genic boundary assignment (5kb upstream, 1.5 kb downstream), whilst the right-panel denotes MAGMA GSA using liberal genic boundary assignment (35 kb upstream, 10 kb downstream). (**c**) A phenome-wide Mendelian randomisation (MR-pheWAS) of genetically predicted *NEK1* decreased mRNA expression using a brain expression quantitative trait loci (eQTL). Each point is the -log10 transformed *P*-value of MR estimates (Wald ratio method statistical method). Clinical endpoints sourced from FinnGen release 10 (N = 412,181), with the horizontal dotted line indicative of Bonferroni significance. Only points with at least a nominal marginal association of the eQTL effect allele with the outcome are plotted.

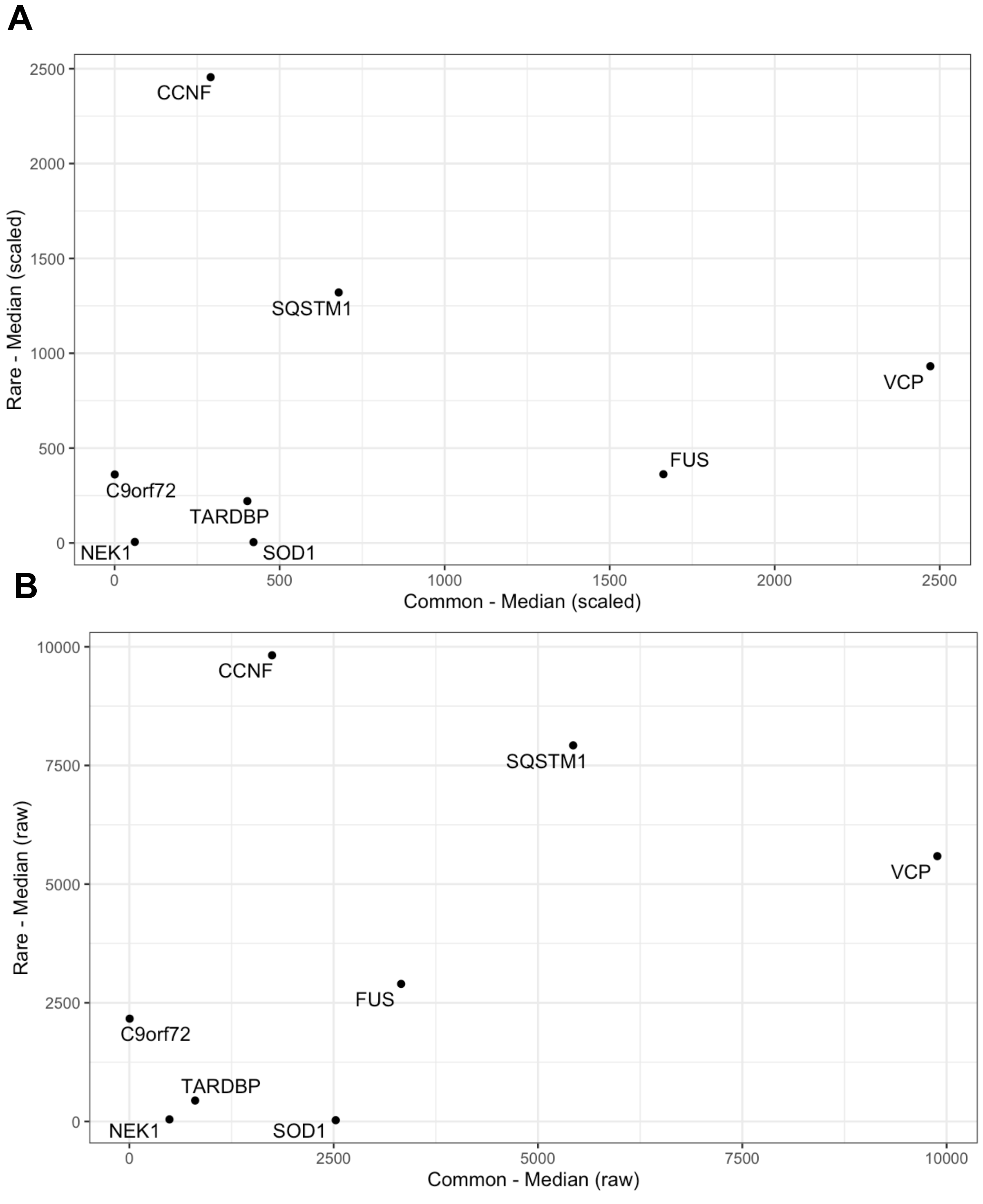

Supplementary Figure 9. Key genes implicated in familial modes of amyotrophic lateral sclerosis (ALS) inheritance with respect to our common and rare-variant led ranking approach. Genes reported as attributable to at least 1% of familial ALS cases plotted here. Each data point denotes a rank: (**a**) scaled median ranks, (**b**) raw median ranks. *NEK1* = NIMA related kinase 1, *CCNF* = cyclin F, *C9orf72* = Chromosome 9 open reading frame 72, *TARDBP* = TAR DNA-binding protein 43, *SOD1* = superoxide dismutase 1, *FUS* = Fused in sarcoma, *SQSTM1* = Sequestosome-1, *VCP* = Valosin containing protein.

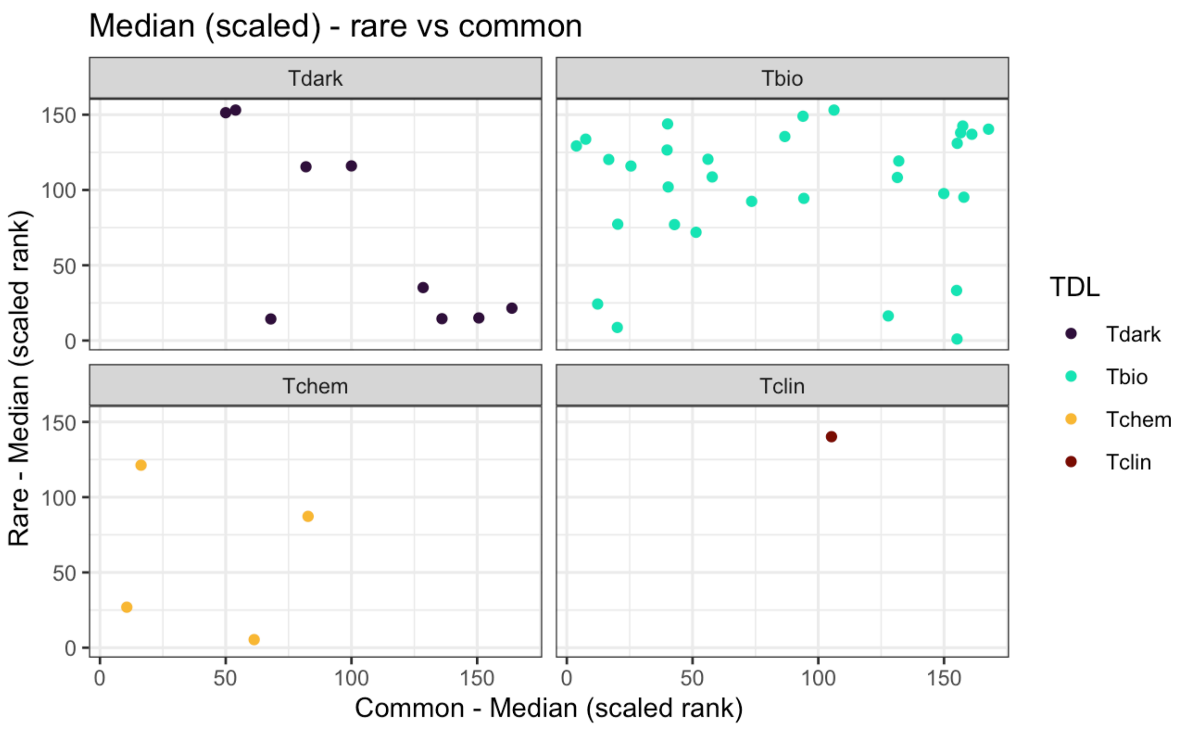

Supplementary Figure 10. Target Central Resource Database (TCRD) therapeutic development level (TDL) annotations for genes in the top 5% of scaled median ranks for both the common and rare-variant led approach. Each data point denotes a rank - rare variant ranks derived from exome-wide association study (ExWAS) with sample size of 6,538 ALS cases, 2,415 controls), whilst common-variant ranks were derived from genome-wide association study (27,205 ALS cases and 110,881 controls). The therapeutic development level (TDL) categories can be conceptualised as follows: T_{clin} denotes genes with an approved drug targeting them, Tchem genes have strong small-molecule binding affinity, T_{bio} genes do not have drug or small molecule activities sufficient to classify them as T_{chem} but are annotated with a gene-ontology molecular function or biological function term with experimental evidence, and T_{dark} are genes which are poorly characterised by both database and literature (text-mined) sources.

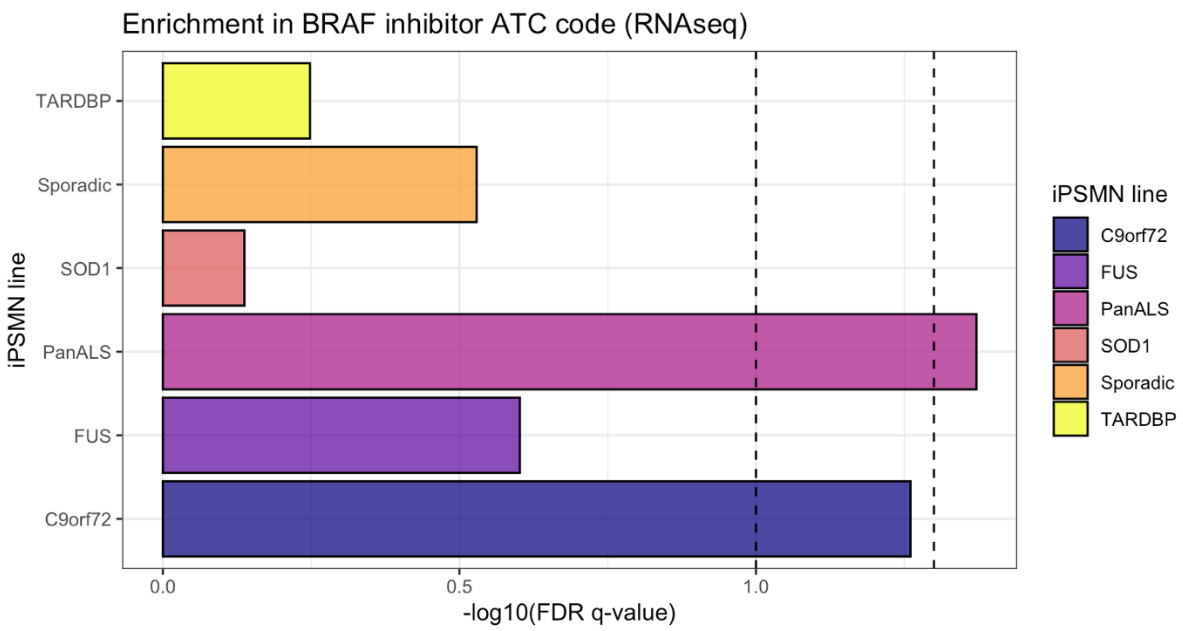

Supplementary Figure 11. Enrichment of ALS associated gene-expression signals amongst targets of BRAF inhibitors in different amyotrophic lateral sclerosis (ALS) induced pluripotent stem cell derived motor neuron line. Rank-based enrichment (GSEApreranked) of genes within the BRAF inhibitor level 4 ATC code derived from their differential expression *P*-value from induced pluripotent stem cell derived motor neuron lines (iPSMN) iPSMN RNA-sequencing based analyses, as outlined in Ziff *et al.* (N = 429). Differential expression analyses were conducted for the following ALS case subgroups versus controls: sporadic, *C9orf72* pathogenic variant carriers, *FUS* pathogenic variant carriers, *SOD1* pathogenic variant carriers, *TARDBP* pathogenic variant carriers, as well as a 'Pan-ALS' mega-analysis of all of the above. The *x*-axis denotes the $-\log_{10}$ transformed FDR *q*-value of the GSEApreanked analysis considering all level 4 ATC codes. The first left-most dotted line is indicative of suggestive corrected significance (FDR $q < 0.1$), whilst the right-most dotted line denotes FDR $q < 0.05$, our multiple-testing correction threshold.

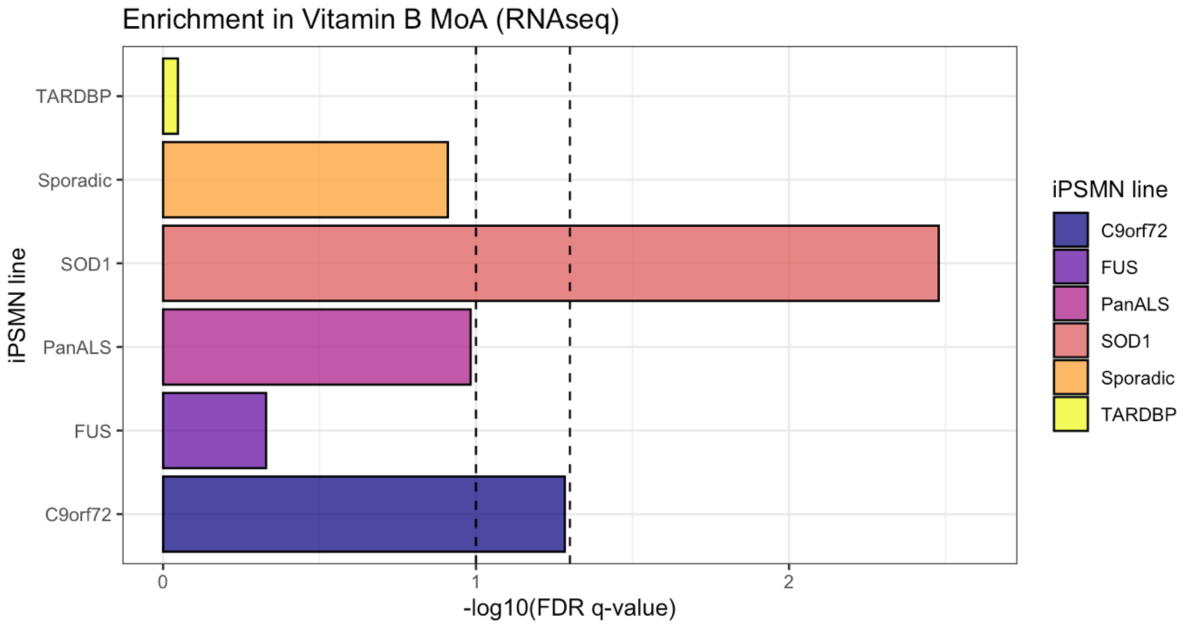

Supplementary Figure 12. Enrichment of ALS associated gene-expression signals amongst targets of the B-vitamin mechanism of action group targets in different amyotrophic lateral sclerosis (ALS) induced pluripotent stem cell derived motor neuron lines. Rank-based enrichment (GSEApreranked) of genes within the B vitamin mechanism of action group targets derived from their differential expression *P*-value from induced pluripotent stem cell derived motor neuron lines (iPSMN) RNA-sequencing based analyses as outlined in Ziff *et al.* (N = 429). Differential expression analyses were conducted for the following ALS case subgroups versus controls: sporadic, *C9orf72* pathogenic variant carriers, *FUS* pathogenic variant carriers, *SOD1* pathogenic variant carriers, *TARDBP* pathogenic variant carriers, as well as a 'Pan-ALS' mega-analysis of all of the above. The x-axis denotes the -log10 transformed FDR q-value of the GSEApreanked analysis considering all MoA groups. The first left-most dotted line is indicative of suggestive corrected significance (FDR q < 0.1), whilst the right-most dotted line denotes FDR q < 0.05, our multiple-testing correction threshold.

SUPPLEMENTARY REFERENCES

1. de Leeuw CA, Mooij JM, Heskes T, Posthuma D. MAGMA: generalized gene-set analysis of GWAS data. *PLoS Comput Biol*. 2015;11(4):e1004219. doi:10.1371/journal.pcbi.1004219

2. Li A, Liu S, Bakshi A, et al. mBAT-combo: A more powerful test to detect gene-trait associations from GWAS data. *Am J Hum Genet*. 2023;110(1):30-43. doi:10.1016/j.ajhg.2022.12.006

3. Liu Y, Xie J. Cauchy Combination Test: A Powerful Test With Analytic *p* -Value Calculation Under Arbitrary Dependency Structures. *J Am Stat Assoc*. 2020;115(529):393-402. doi:10.1080/01621459.2018.1554485

4. Wainberg M, Sinnott-Armstrong N, Mancuso N, et al. Opportunities and challenges for transcriptome-wide association studies. *Nat Genet*. 2019;51(4):592-599. doi:10.1038/s41588-019-0385-z

5. Gandal MJ, Zhang P, Hadjimichael E, et al. Transcriptome-wide isoform-level dysregulation in ASD, schizophrenia, and bipolar disorder. *Science*. 2018;362(6420). doi:10.1126/science.aat8127

6. Zhu Z, Zhang F, Hu H, et al. Integration of summary data from GWAS and eQTL studies predicts complex trait gene targets. *Nat Genet*. 2016;48(5):481-487. doi:10.1038/ng.3538

7. Giambartolomei C, Vukcevic D, Schadt EE, et al. Bayesian test for colocalisation between pairs of genetic association studies using summary statistics. *PLoS Genet*. 2014;10(5):e1004383. doi:10.1371/journal.pgen.1004383

8. van Rheenen W, van der Spek RAA, Bakker MK, et al. Common and rare variant association analyses in amyotrophic lateral sclerosis identify 15 risk loci with distinct genetic architectures and neuron-specific biology. *Nat Genet*. 2021;53(12):1636-1648. doi:10.1038/s41588-021-00973-1

9. Raudvere U, Kolberg L, Kuzmin I, et al. g:Profiler: a web server for functional enrichment analysis and conversions of gene lists (2019 update). *Nucleic Acids Res*. 2019;47(W1):W191-W198. doi:10.1093/nar/gkz369

10. Reay WR, Geaghan MP, 23andMe Research Team, et al. The genetic architecture of pneumonia susceptibility implicates mucin biology and a relationship with psychiatric illness. *Nat Commun*. 2022;13(1):3756. doi:10.1038/s41467-022-31473-3

11. Reay WR, Kiltschewskij DJ, Di Biase MA, et al. Genetic influences on circulating retinol and its relationship to human health. *Nat Commun*. 2024;15(1):1490. doi:10.1038/s41467-024-45779-x
